# K-Ion Slides in Prussian Blue Analogues

**DOI:** 10.1021/jacs.3c08751

**Published:** 2023-10-25

**Authors:** John Cattermull, Nikolaj Roth, Simon J. Cassidy, Mauro Pasta, Andrew L. Goodwin

**Affiliations:** † Inorganic Chemistry Laboratory, Department of Chemistry, University of Oxford, South Parks Road, Oxford OX1 3QR, U.K.; ‡ Department of Materials, University of Oxford, Parks Road, Oxford OX1 3PH, U.K.; ¶ iNANO, Aarhus, DK-8000 Denmark

## Abstract

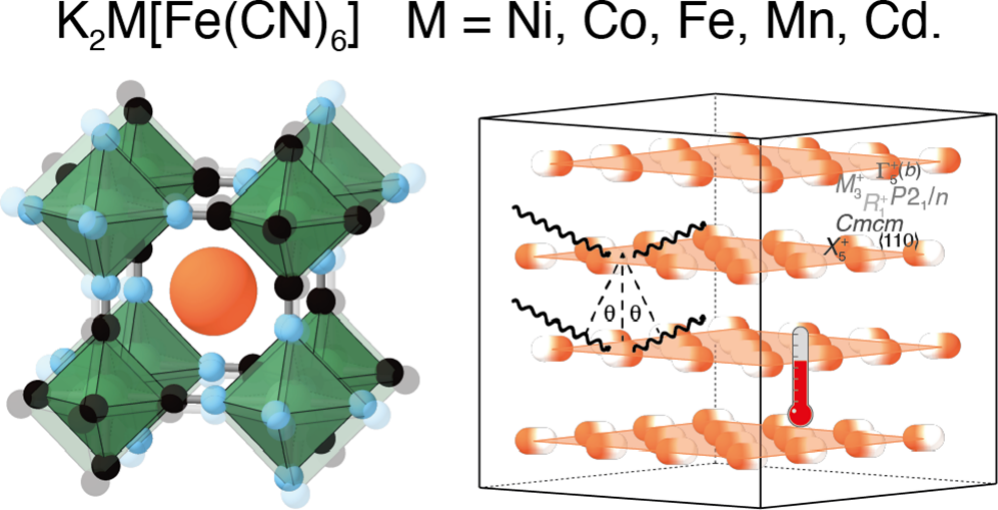

We study the phenomenology of cooperative off-centering of K^+^ ions in potassiated Prussian blue analogues (PBAs). The principal distortion mechanism by which this off-centering occurs is termed a “K-ion slide”, and its origin is shown to lie in the interaction between local electrostatic dipoles that couple through a combination of electrostatics and elastic strain. Using synchrotron powder X-ray diffraction measurements, we determine the crystal structures of a range of low-vacancy K_2_M[Fe(CN)_6_] PBAs (M = Ni, Co, Fe, Mn, Cd) and establish an empirical link between composition, temperature, and slide-distortion magnitude. Our results reflect the common underlying physics responsible for K-ion slides and their evolution with temperature and composition. Monte Carlo simulations driven by a simple model of dipolar interactions and strain coupling reproduce the general features of the experimental phase behavior. We discuss the implications of our study for optimizing the performance of PBA K-ion battery cathode materials and also its relevance to distortions in other, conceptually related, hybrid perovskites.

## Introduction

Prussian blue analogues (PBAs) are a historically important family of inorganic framework materials, known for their gas-storage, catalytic, photophysical, magnetic, and electrochemical properties.^1−5^ In contemporary language, PBAs are a kind of hybrid perovskite:^6^ their structure is based on that of the cubic ABX_3_ perovskite net, with cyanide molecules playing the role of the X^–^ anionic component. One consequence of this molecular building block is that the perovskite cage, i.e., the volume surrounding A^+^ cations, is significantly larger than that in conventional perovskite ceramics, and this leads to a greater flexibility of the anionic framework.^7,8^ This flexibility can be exploited in anomalous mechanical properties such as negative thermal expansion,^9,10^ but it also predisposes PBAs to structural distortions in response to compositional variation^11^ and/or external stimuli, such as temperature or pressure.^12,13^ If there is one key lesson from the structural chemistry of conventional perovskites it is that developing control over the presence and nature of distortion mechanisms is essential in functional materials design.^14^ The “tilt-engineering” approach^15−18^ responsible for stabilizing simultaneous electric polarization and bulk magnetism at room temperature in the layered perovskites (Ca_*y*_Sr_1–*y*_)_1.15_Tb_1.85_Fe_2_O_7_ is a high-profile example.^19^ It is in this context that there has been substantial recent interest in the relevance of structural distortions for the physical and chemical properties of PBAs.^8,20−23^

One domain of PBA chemistry in which distortions appear particularly important is the application of PBAs as cathode materials for Na- and especially K-ion batteries.^8,23,24^ PBAs are arguably the most promising cathode material for K-ion batteries, identified for their high operating voltages, favorable charge rates, and inexpensive solution-phase synthesis from earth-abundant elements.^25,26^ The specific capacity of PBAs—general formula A_*x*_B[B′(CN)_6_]_*y*_—depends not only on the atomic weight and redox properties of the transition-metal ions B and B′ but also on the concentration of hexacyanometallate vacancies. Efforts to increase the specific capacity of PBA cathodes by reducing vacancy concentrations (i.e., *y* → 1) have resulted in materials that exhibit structural phase changes on electrochemical cycling.^26^ Despite the increased capacity being realized, the rate capability of low-vacancy PBAs is significantly diminished and can only be recovered by cycling much smaller particles, which degrade faster over time.^27^ Multiphase behavior on cycling is normally associated with poor rate performance in electrodes.^28,29^ The driving force responsible for this phase-change behavior is not yet well understood, but the empirical observation is that stoichiometric A_*x*_B[B′(CN)_6_] compounds distort away from the parent cubic perovskite-like structure at high concentrations of the A-site cation 1 ≤ *x* ≤ 2.^8,30^ Hence the optimization of PBA cathode performance involves establishing control over this symmetry-lowering process, through understanding its interplay with PBA composition.

**Figure 1:**
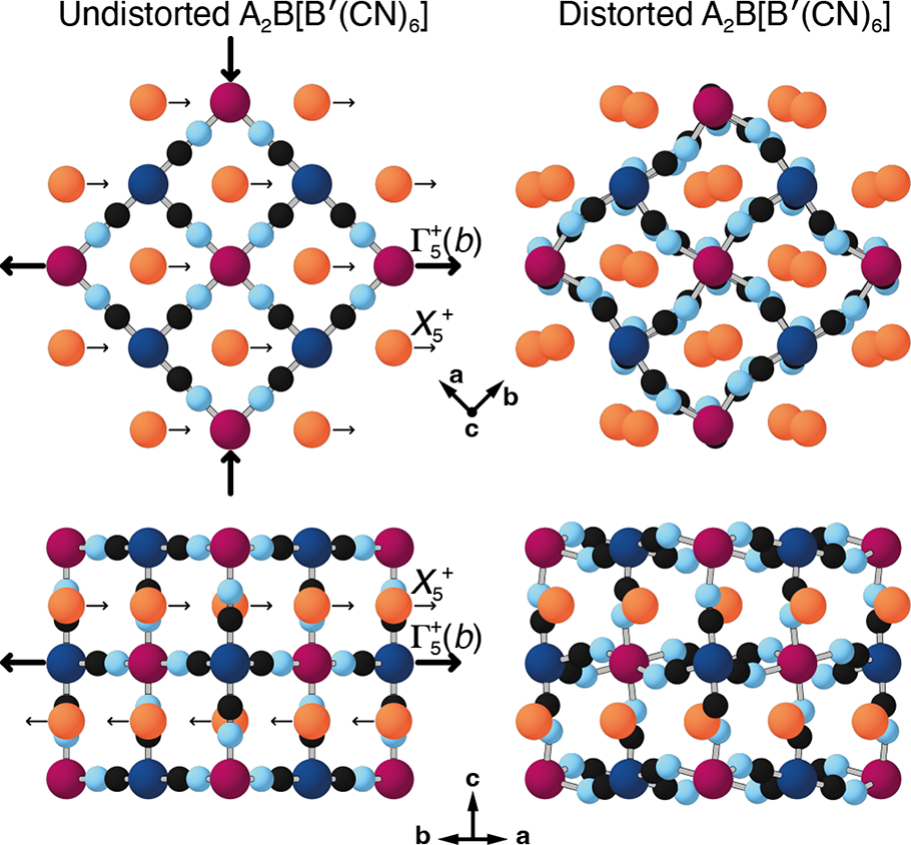
Representation of slide distortion in K-rich PBAs and its coupling to strain. K^+^ ions (orange spheres) occupy the A-site positions of the perovskite-like PBA structure and translate in a direction parallel to one of the ⟨110⟩ axes. Neighboring layers alternate displacement direction. The views here are given normal (top) and parallel (bottom) to the K-ion layers. The Γ_5_^+^(*b*) strain mode describes wine-rack-like flexing (bold arrows) of the PBA framework as the slide is activated. B, B′, C, and N atoms are shown as magenta, dark blue, black, and light blue spheres, respectively.

Structural distortions of PBAs come in a number of different flavors.^7,8,31^ As perovskite analogues, the distortions found in conventional ABX_3_ materials—octahedral tilts,^32,33^ cooperative Jahn–Teller order,^34,35^ A-site or B-site compositional order^36^—are all observed in various PBAs.^8^ Likewise, the additional framework degrees of freedom allowed in hybrid perovskites—forbidden tilts^37^ and columnar shifts^38^—also play a role in static and dynamic distortions of PBA structures, respectively. But the lowest-energy (and hence most physically important) distortion mechanism in the K-rich PBA cathode materials involves cooperative off-centering of K^+^ ions within the cube-like A-site cavities toward an edge of the surrounding anionic PBA framework [Figure 1].^8,39^ The displacements couple such that cations “slide” together as layers along a common ⟨110⟩ direction; this direction alternates from layer to layer. Whereas in conventional perovskites A-site off-centering is often driven by second-order Jahn–Teller effects and results in relatively small displacements,^40,41^ in K-rich PBAs the driving force is electrostatic and the displacements large (≃ 0.5 Å). We showed recently that, in K_2_Cu[Fe(CN)_6_], K-ion slides are switched off when the material is heated and also when K^+^ is extracted electrochemically.^39^ Beyond this example, however, there is no clear understanding of the general phenomenology of K-ion slides in PBAs or how the distortion might be controlled by varying the PBA composition.

In this study, we use a combination of group-theoretical arguments, simulations, and crystallographic measurements to probe both the origin of K-ion slides and also their interplay with variation in temperature and PBA composition. Our calculations are based on a simple model of local electrostatic dipoles that couple through a combination of long-range dipolar interactions and elastic strain. We show that this model can account not only for the particular pattern of A-site displacements associated with K-ion slides but also for the disappearance of the distortion at moderate elevated temperatures. Experimentally, we determine the crystal structures of a range of K_2_M[Fe(CN)_6_] PBAs (M = Ni, Co, Fe, Mn, Cd) and establish an empirical link between composition and slide-distortion magnitude that reflects the expectations of our toy model. Using variable-temperature X-ray diffraction measurements, we determine the thermal response of the K-ion slides as a function of composition. We show that our results reflect a common underlying physics responsible for K-ion slides and their evolution with temperature and composition, based on the dipolar interactions of our toy model and a simple linear relationship between PBA framework size and degree of K-ion off-centering. Our paper concludes with a discussion of the implications of our study for optimizing PBA battery materials and also with a more general discussion of the role of slide distortions in other hybrid perovskite materials.

## Theory

### Group-Theoretical Description of K-Ion Slides

We begin with a formal definition of K-ion slides in PBAs, drawing on the group-theoretical language of irreducible representations (irreps).^42^ In this context, the K-ion slide distortion illustrated in Figure 1 is associated with the *X*_5_^+^ irrep of the perovskite ABX_3_ structure type, with its order parameter polarized along ⟨110⟩. (Here we are using the convention of placing the A-site at the center of the *Pm*3̅*m* unit-cell; a different choice of origin will change the irrep labels given here, but not the underlying symmetry arguments.) By itself, this distortion reduces the *Pm*3̅*m* crystal symmetry of the parent aristotype to an orthorhombic *Cmcm* supercell. K-rich PBAs also support a set of octahedral tilts that act to reduce the framework volume and increase electrostatic interactions between the K^+^ A-site cations and the anionic framework.^21^ The dominant tilt system in PBAs is characterized by the *M*_3_^+^ irrep polarized along ⟨100⟩; this tilt has *a*^0^*a*^0^*c*^+^ Glazer notation and corresponds to in-phase tilts along the cube axis perpendicular to the slide planes.^32,43^ Crystal symmetry is further lowered by the alternation of M and M′ transition-metal ions on the B-site of the perovskite lattice; this decoration reduces crystal symmetry in a way that is captured by the *R*_1_^+^ irrep. Taken together, the combination of *X*_5_^+^ K-ion slides, *M*_3_^+^ tilts, and *R*_1_^+^ B-site order collectively reduces the crystal symmetry of K-rich PBAs to the monoclinic space group *P*2_1_/*n*, which is almost universally observed experimentally.^8,21^

The degree of monoclinic distortion is related to the magnitude of the K-ion slides. In the absence of any slide distortion, the combination of octahedral tilts and B-site cation order gives tetragonal crystal symmetry with space group *P*4/*mnc*. While the primary order parameter associated with K-ion slides is the *X*_5_^+^ irrep described above, a secondary order parameter that couples to the slides is the monoclinic strain, described by the Γ_5_^+^(*b*) irrep. This strain distortion can be understood as a flexing of the cubic unit cell, stretching along the particular diagonal direction in which the K-ions displace, while contracting in a perpendicular direction [Figure 1]. In crystallographic experiments, this distortion has a clear signature in the splitting of specific reflections, and so the thermal evolution of K-ion slide displacements can often be inferred directly by interrogation of the variable-temperature diffraction pattern.^39^ We will return to this point in due course.

Activation of the K-ion slides within the tilted B-site-ordered PBA structure induces a large number of additional secondary order parameters beyond those already discussed here. We include in the Supporting Information a complete description of these secondary distortion modes but comment briefly on two of these. One is an additional tilt system with Glazer notation *a*^0^*b*^–^*b*^–^ (irrep *R*_4_^+^) which is polarized along a direction perpendicular to the primary tilt axis. Collectively these two tilts combine to give the *a*^–^*a*^–^*b*^+^ tilt pattern that has been noted elsewhere for monoclinic PBAs.^21^ The other secondary distortion that we discuss involves a component of the K-ion displacements. While the slide distortion polarized along ⟨110⟩ dominates displacements away from the high-symmetry A-site, in the presence of the tilts, there is an additional component along ⟨100⟩ characterized by the *R*_5_^+^ irrep that is introduced. We will discuss this additional K-ion displacement in the context of the high-temperature structures of K-rich PBAs.

### Dipolar Stabilization of K-Ion Slides

Having summarized the empirical form of K-ion slides in K-rich PBAs, we proceed to address the important question of *why* this particular pattern of A-site off-centering is actually observed in practice. We take as our starting point the assumption that electrostatic interactions and steric considerations conspire to favor off-centering of each individual K^+^ ion within the anionic cubic cage in which it lies and that the type of direction is the same from cage to cage. We then consider, from a group-theoretical perspective, the various ways in which this A-site off-centering can couple to break the crystal symmetry of the *Pm*3̅*m* ABX_3_ perovskite aristotype. In Table 1 we enumerate^44,45^ the simplest such couplings, by which we mean the distortions satisfying three criteria: (i) the A-site displacements **e** are polarized along one of the high-symmetry directions ⟨100⟩, ⟨110⟩, ⟨111⟩ and are constrained by symmetry to do so; (ii) the modulation wave-vector(s) **k** correspond(s) to the high-symmetry points Γ, *X*, *M*, *R*; and (iii) all A-site displacements within a given distortion are constrained by symmetry to have the same magnitude. The zone-center modes—all of which are characterized by the same irrep Γ_4_^–^—are the familiar polar distortions discussed elsewhere in the context of ferroelectric and multiferroic perovskites.^46^ The remaining distortion modes are all nonpolar.

**Table 1:**
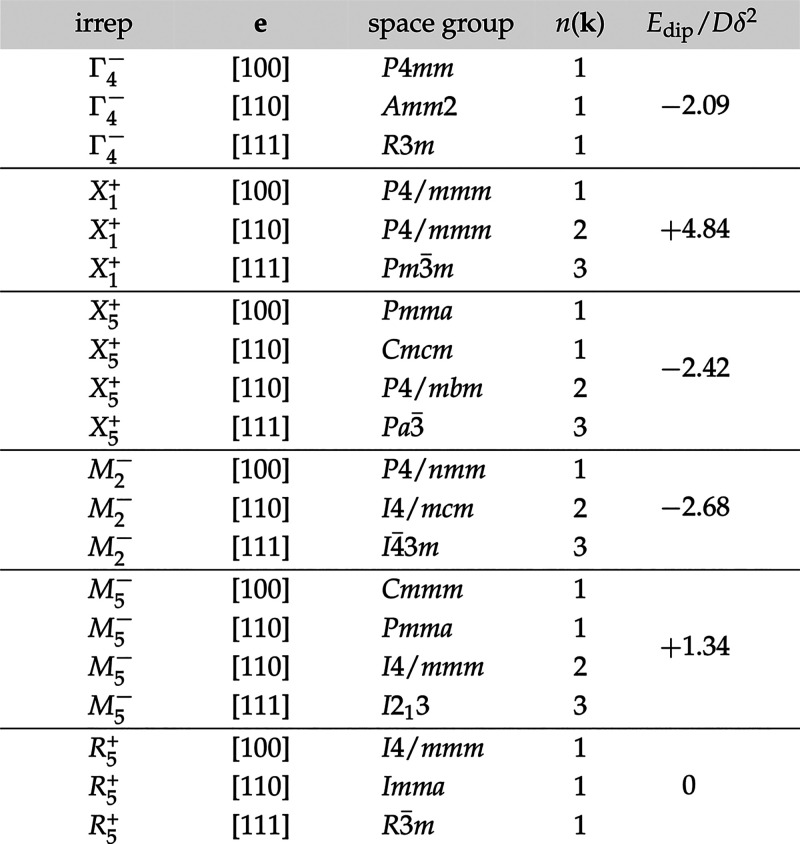
List of Simplest A-Site Off-Centering Distortion Modes, Grouped According to the Corresponding Irrep^a^

a *n*(**k**) denotes the number of **k**-vectors involved in the distortion mode.

A universal effect of off-centering of the A-site is that an electrostatic dipole is necessarily generated within each unit cell. These dipoles interact with one another via long-range dipolar coupling, to give different contributions to the lattice energy for the different distortion patterns enumerated in Table 1. The dipolar coupling energy can be determined as
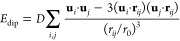
1where the sum is taken over all unit cells *i*, *j* separated by vector **r**_*ij*_, with **u**_*i*_ = δ**e**_*i*_ being the displacement vector of magnitude δ and polarization **e**_*i*_ within unit cell *i*, *D* the dipolar coupling strength, and *r*_0_ the nearest-neighbor dipole separation. Using the Ewald summation code developed in ref (47), we calculated the normalized dipolar coupling energy for all displacement modes in Table 1. Based on dipolar interactions alone, the most stable A-site displacement pattern is that characterized by the *M*_2_^–^ irrep,^48^ which is different from the slide distortion observed experimentally.

Included in our group-theoretical enumeration are a number of multi-**k** distortion modes since these satisfy the various criteria given above. In each case, however, the actual A-site displacements occur along different axes (albeit of the same type) in neighboring cells. For example, the 2-**k***M*_2_^–^ distortion polarized along ⟨110⟩ gives an *I*4/*mcm* structure with displacements along four directions [±1, ±1, 0]. Since the K-ion displacements couple strongly to a flexing of the anionic lattice, we anticipate that single-**k** distortions—for which all A-site displacements occur along a single axis—are stabilized relative to multi-**k** distortions through coupling to strain. For displacements along the ⟨110⟩ directions, the lowest-energy single-**k** coupling is indeed the *X*_5_^+^ irrep that describes K-ion slides in PBAs.

So our interpretation is that the polarization direction is a function of the local chemistry, i.e., selected so as to maximize interactions between the K^+^ ion and its surrounding anionic framework. If the coupling between off-centering directions in one unit cell and its neighbors is driven by a combination of dipole–dipole interactions and strain coupling, then the *X*_5_^+^ slide distortion is the lowest-energy arrangement of these displacements. We suggest this is why this particular slide distortion is so common among K-ion PBAs.

### Toy Microscopic Model

What follows from this group-theoretical analysis is a simple microscopic model for the interactions from which K-ion slides emerge. Combining the considerations of strain coupling, on the one hand, and dipole–dipole interactions, on the other hand, we arrive at the effective Hamiltonian:
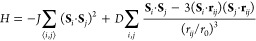
2which we have based on the models studied in refs (49)–^51^. In this picture, we treat A-site displacements as effective pseudospins  of unit length (formally, a 12-site Potts model). The dipolar term in eq 2 is exactly that given in eq 1, and we add to this a simplified strain-coupling term,^49,51^ which acts to align A-site displacement axes in neighboring cells (denoted here by ⟨*i*, *j*⟩). For appropriate ratios *D*/*J* (i.e., when *J* is not too small), the ground state of eq 2 has antipolar order of the kind associated with cooperative K-ion slides.^51^

**Figure 2:**
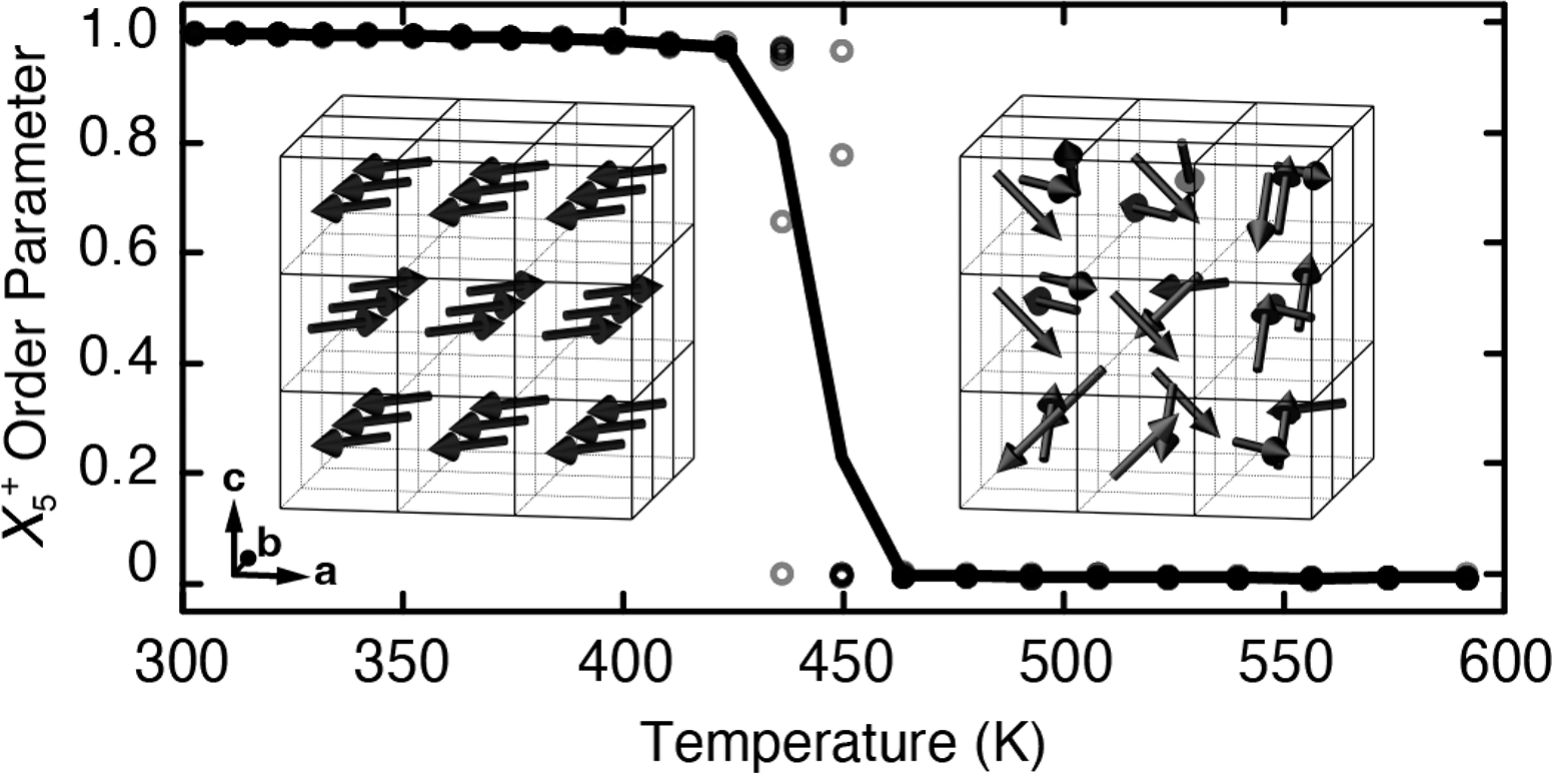
Temperature-dependence of K-ion slide order in Monte Carlo simulations driven by eq 2, parametrized as discussed in the text. Data points (open circles) are shown for eight independent Monte Carlo simulated annealing trajectories. The solid line tracks the thermal evolution of the order parameter, evaluated as the average over these eight runs.

We illustrate this point in Figure 2, where we plot the temperature dependence of the *X*_5_^+^ order parameter extracted from Monte Carlo simulations driven by eq 2 with *J* = *D*. An order/disorder transition is observed with critical temperature *T*_c_ ≃ 1.4*D* for temperatures above this value, and one expects the slide distortion to disappear and A-site displacements to become uncorrelated. (Note that the transition temperature is largely independent of *J*, which acts only to select the single-**k** ground state. In the study of ref (51), it was found that a critical value of *J* was given by 0.22*D*, at which point the ground state switches from multi-**k** to single-**k**.) We have placed these simulations on an absolute energy scale by calculating the magnitude of *D* from estimates of the degree of A-site off-centering and the spacing between neighboring dipoles:
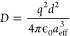
3Here *q* is the A-site ion charge and *d* the A-site displacement magnitude. Using a typical value *d* = 0.5 Å (e.g., as observed in K_2_Mn[Fe(CN)_6_]) and an effective PBA cell parameter *a*_eff_ of 5 Å, one obtains *D* ≃ 330 K, and hence *T*_c_ ≃ 460 K. So this simple toy model anticipates the thermal deactivation of tilts at moderately elevated temperatures, e.g., as observed previously for K_2_Cu[Fe(CN)_6_] (for which *T*_c_ ≃ 520 K).^39^ We will return to this point in due course.

One implication of this result, which deserves brief comment, concerns the identification of K-ion slides as the primary order parameter for this transition. From a group-theoretical perspective, the transition might equally well be associated with switching on or off, for example, the secondary *R*_4_^+^ tilt system;^21,39^ there are other possible primary order parameter choices. It is meaningless of course to disentangle the various effects, since they are all coupled by symmetry, but the key point here is that the dipolar interactions from which the slides arise have the appropriate energy scale to rationalize the transition temperature observed in practice.

## Results and Discussion

### Compositional Dependence of the Slide Distortion

**Figure 3:**
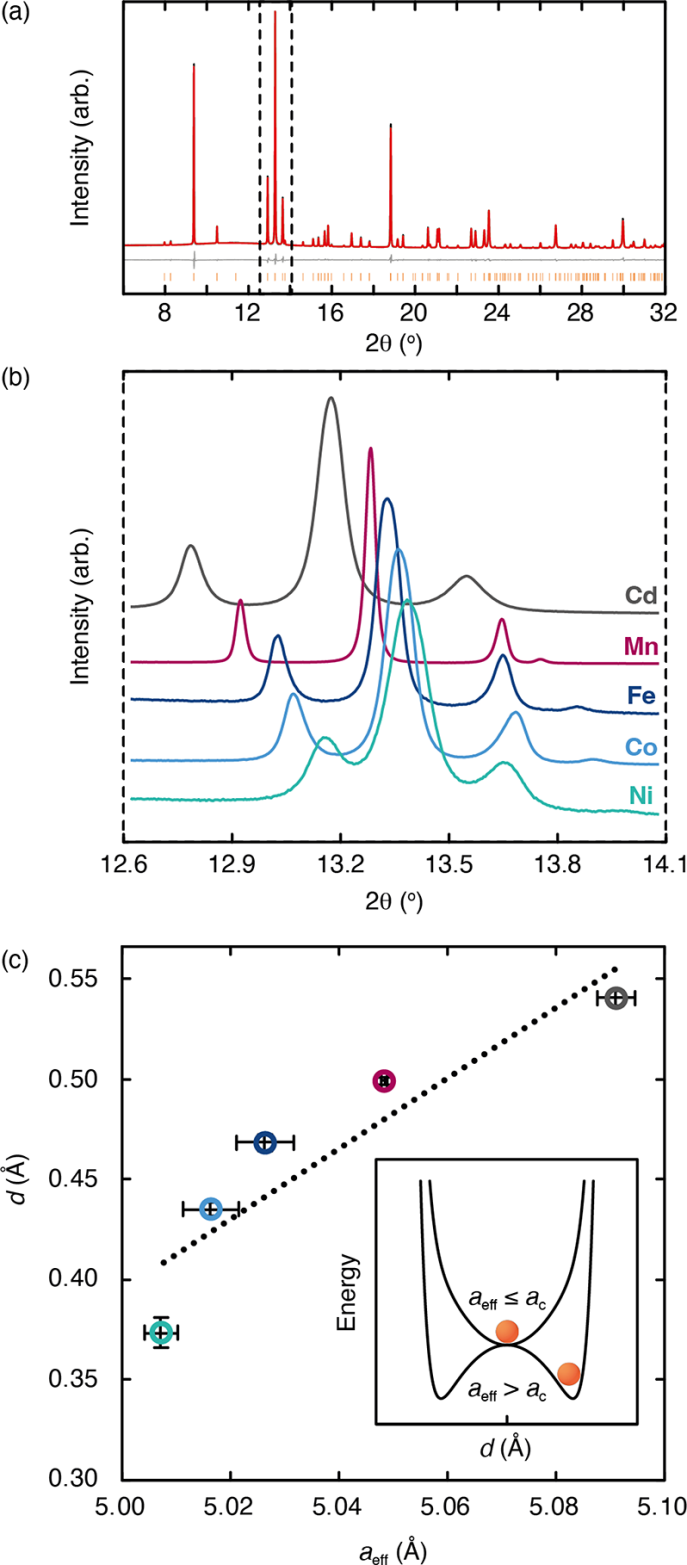
(a) Room-temperature synchrotron powder X-ray diffraction pattern and the corresponding Rietveld fit for K_2_Mn[Fe(CN)_6_]. Data are shown in black, fit is shown in red, difference curve is shown in gray (data-fit) offset below the data, and reflection positions are shown as orange tick marks. (b) Compositional variation of the X-ray diffraction patterns of K_2_M[Fe(CN)_6_] samples in the region related to the (110) reflection of the parent PBA aristotype. The splitting seen here, which is between the , and (002) reflections, is characteristic of the degree of monoclinic strain Γ_5_^+^(*b*). (c) Relationship between K-ion displacements *d* and effective unit-cell constant *a*_eff_ in K_2_M[Fe(CN)_6_], with colors denoting M as indicated in (b). The inset schematically shows the proposed variation in local effective potential at the A-site as *a*_eff_ is varied.

Such is the diversity of accessible PBA compositions that we were able to synthesize a range of samples by varying the transition-metal cation on the B-site. Low-vacancy samples of composition K_2_M[Fe(CN)_6_] (M = Ni, Co, Fe, Mn, Cd) were prepared following the methodology of ref (39), and their crystal structures interrogated using synchrotron X-ray powder diffraction measurements. We show in Figure 3(a) a representative ambient-temperature diffractogram; here that was obtained for M = Mn. The diffraction patterns measured for other compositions are essentially identical (see the Supporting Information), but with small variations in peak positions and splittings, as discussed in further detail below. In all cases, we observed sharp reflections characteristic of highly crystalline samples, which is also clear from the scanning electron micrographs shown in the Supporting Information. The peaks observed in each measurement could be indexed according to the *P*2_1_/*n* space group of K_2_Mn[Fe(CN)_6_];^26^ no impurity phases were found. No difference of peak shape was detected in the superlattice reflections, which is consistent with the slide distortion being long-range in the cyanide network.

Systematic trends can be observable directly in the raw data themselves. In Figure 3(b) we show the compositional dependence of the diffraction behavior near the position associated with the (110) reflection of the aristotypic  unit cell. In this region, one observes a dominant reflection flanked by two smaller reflections on either side. The degree of splitting in this triplet of peaks is directly related to the magnitude of monoclinic strain in the *P*2_1_/*n* unit cell. Two systematic trends are obvious. First, there is a shift toward higher reflection angles, i.e., smaller cell volumes, as the M^2+^ ionic radius decreases. And, second, the degree of monoclinic strain also decreases with decreasing M^2+^ size.

In order to quantify these trends and to understand in detail the structural variations effected by changing PBA composition, we carried out Rietveld refinements of each diffraction measurement. We used a distortion-mode approach^44,45^ as implemented in the TOPAS software to do so.^52^ High-quality fits were obtained for all five measurements (see Figure 3(a) for M = Mn and the Supporting Information for other compositions), and we were able to use these refinements to test the degree of K-ion occupancy (itself linked to hexacyanometallate vacancy fraction), obtaining compositions K_*x*_M[Fe(CN)_6_] with 1.95 < *x* ≤ 2 in all cases. The high K-ion concentrations for these samples were further probed by elemental analysis reported in the Supporting Information. Accordingly, we assume hereafter the stoichiometric *x* = 2 composition. Key details of our crystallographic models are summarized in Table 2, where we have included three derived parameters: the effective PBA cell constant, the K-ion displacement magnitude *d*, and the corresponding dipolar coupling strength *D* calculated using eq 3. A full list of refined parameters and their values is provided in the Supporting Information. The two trends already identified in the raw data are supported by the results of our refinements: both *a*_eff_ and the monoclinic strain parameter Γ_5_^+^(*b*) decrease systematically with decreasing transition-metal radius *r*(Cd^2+^) > *r*(Mn^2+^) > *r*(Fe^2+^) > *r*(Co^2+^) > *r*(Ni^2+^).^53^

**Table 2:**
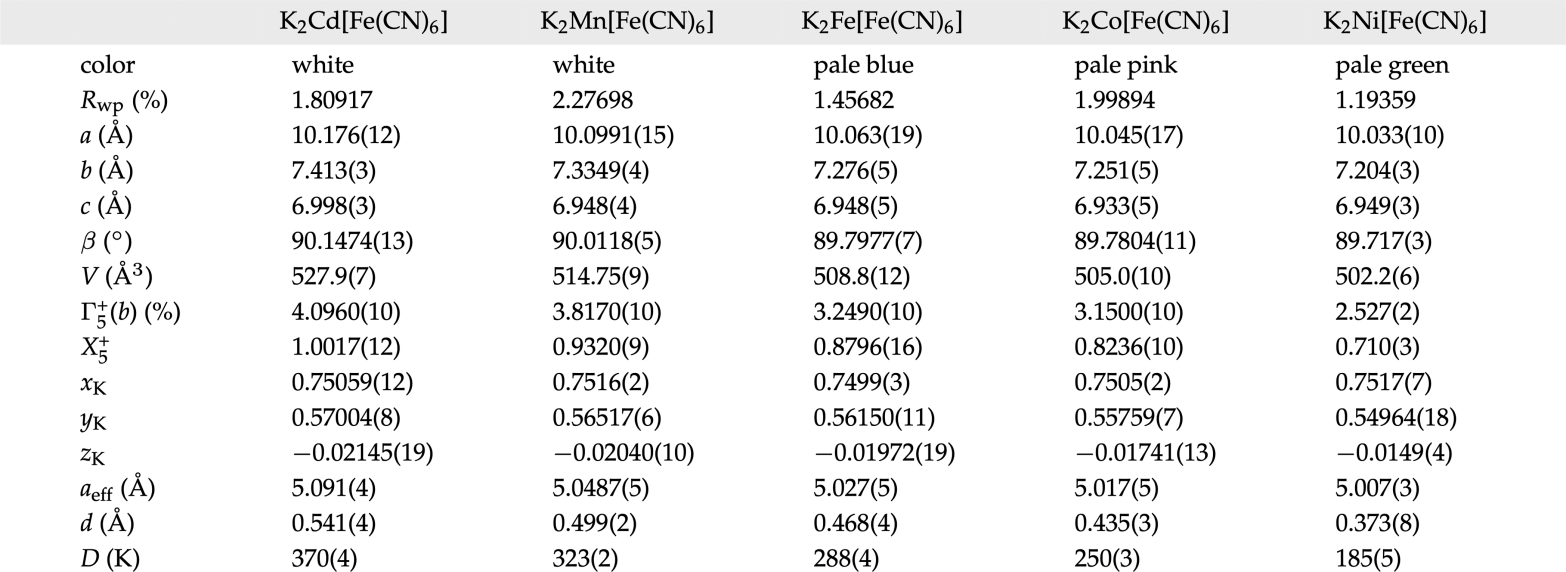
Refinement Statistics, Selected Crystallographic Details, and Derived Parameters Determined for K_2_M[Fe(CN)_6_] Samples Using the Rietveld Refinement of Room-Temperature Synchrotron X-ray Powder Diffraction Measurements

Our refinements allow us to link the degree of slide distortion to PBA composition. We show in Figure 3(c) the variation in K-ion off-centering *d* with the effective PBA unit-cell dimension *a*_eff_. The value of *d* increases rapidly with *a*_eff_: a change of the latter by less than 2% corresponds to a 50% increase in the former. One implication of our data is that the slide distortion should vanish for small PBA cells with *a*_eff_ less than some critical value *a*_c_. Our interpretation of this observation is that the effective potential well in which the K-ion sits has a single minimum at the high-symmetry A-site position for small *a*_eff_, i.e., when the geometric difference in K^+^ effective size and cavity volume is small, but is multiwelled as the cage dimensions increase, and this geometric difference becomes more important. As *a*_eff_ continues to increase beyond *a*_c_, the minimum of the effective potential well shifts further and further away from the high-symmetry A-site, increasing the off-centering magnitude *d* [see inset to Figure 3(c)]. Our data are too sparse for us to determine with any confidence the precise relationship between *d* and *a*_eff_, so we report only the leading-order (linear) approximation
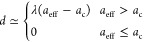
4with empirical parameters λ = 1.75 and *a*_c_ = 4.77 Å, shown as the dashed line in Figure 3(c). Preparation and characterization of solid-solution compositions K_2_(M,M′)[Fe(CN)_6_] would in principle allow a finer mesh of *a*_eff_ values to be explored, and hence a more exact empirical relationship between *a*_eff_ and *d* to be determined.

### Temperature Dependence of the Slide Distortion

We showed in ref (39) that, for the specific material K_2_Cu[Fe(CN)_6_], K-ion slides can be switched off at elevated temperatures. Indeed, as we have already mentioned, the thermal deactivation of slides is intrinsic to the simple dipolar model represented in Figure 2. Consequently we extended our synchrotron X-ray powder diffraction measurements of K_2_M[Fe(CN)_6_] samples to span the temperature range 300 ≤ *T* ≤ 1000 K. We will come to show that we found qualitatively similar behavior for all materials—and hence the sensitivity of K-ion slides to temperature is a general feature of the family—but focus first on describing our observations for K_2_Mn[Fe(CN)_6_], which is representative of the family as a whole.

**Figure 4:**
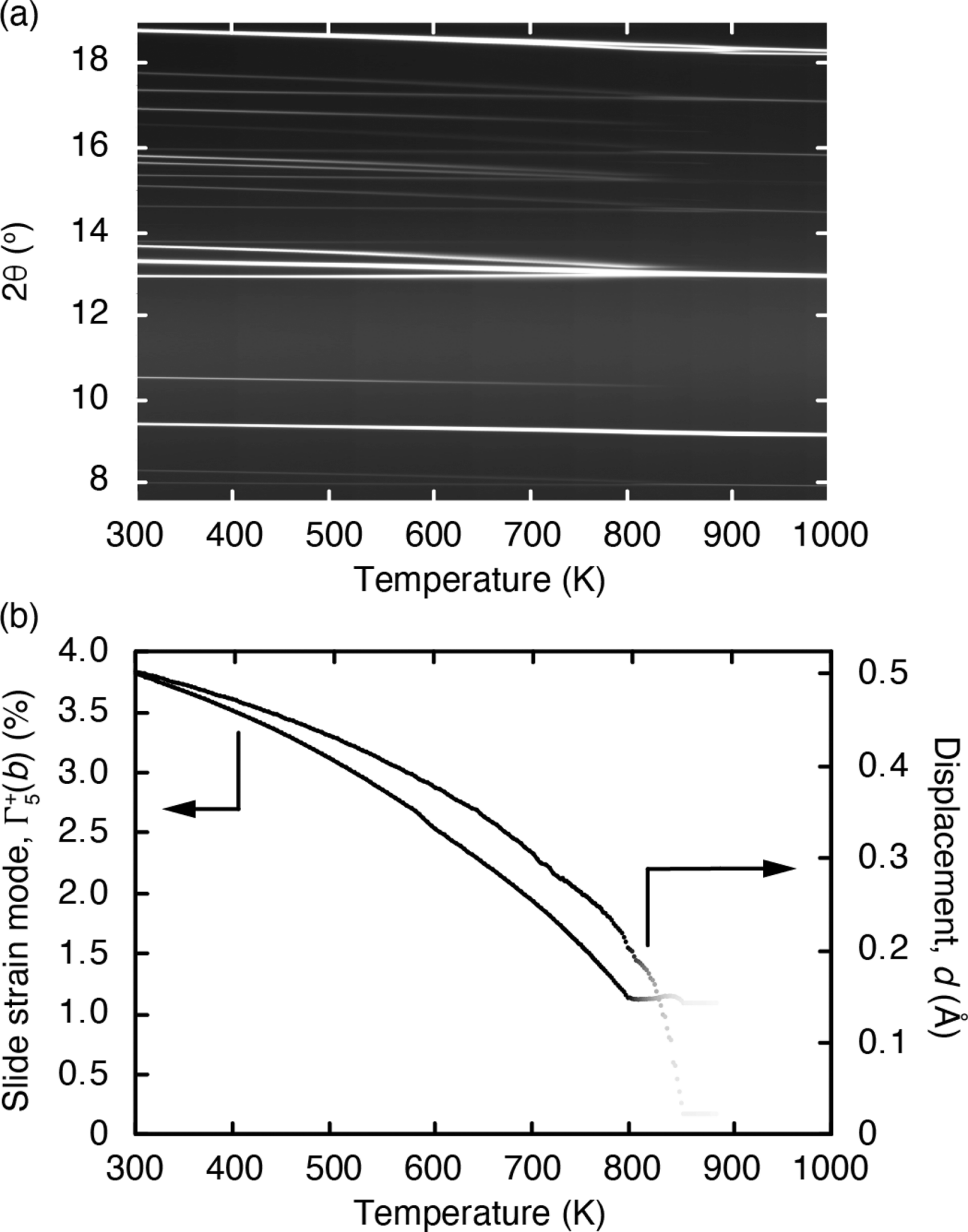
(a) Variable-temperature synchrotron powder X-ray diffraction patterns (λ = 0.825318(3) Å) for K_2_Mn[Fe(CN)_6_], represented as a film plot. The triplet of peaks near 2θ = 13° corresponds to those highlighted in Figure 3(b). Note the presence of phase transitions at *T* ≃ 800 and 900 K. (b) Corresponding thermal variation in slide strain mode Γ_5_^+^ and K-ion displacements *d*, as determined using Rietveld refinement of the diffraction data shown in (a). The intensity of the data points is scaled by the phase fraction of the *P*2_1_/*n* ambient phase to reflect the contribution that the parameters make to the overall refinement of the XRD pattern.

Our experimental measurements are summarized in Figure 4(a), where we have compiled diffractograms measured for K_2_Mn[Fe(CN)_6_] at intervals of 2 K into a combined film plot and have focused on a key region of the diffraction pattern. Clearly visible at low temperatures is the triplet of peaks near 2θ ≃ 13° characteristic of K-ion-slide-driven monoclinic splitting [cf. Figure 3(a,b)]. This splitting becomes systematically less pronounced with increasing temperature until about 800 K, whereafter a number of peaks disappear and the diffraction pattern simplifies considerably. Hence the ambient phase is stable up to a critical temperature *T*_c_ ≃ 800 K.

Distortion-mode Rietveld refinements allow us to track the structural effects of the temperature throughout this ambient-temperature phase. The distortion parameters that show the greatest temperature dependence are the monoclinic strain mode Γ_5_^+^(*b*), on the one hand, and those governing the displacement of K^+^ ions away from the high-symmetry A-site position, on the other hand. We plot in Figure 4(b) the temperature dependence of these two parameters; full details of the refinements are provided in the Supporting Information. What is clear is that heating K_2_Mn[Fe(CN)_6_] rapidly deactivates its K-ion slide distortion, both in the sense that the average position of the K^+^ ions increasingly returns to the center of the cubic cavity in which they are located and also in the degree of macroscopic strain associated with off-centering. The transition observed at 800 K appears to be first order (discontinuous). Because we continued to heat the sample beyond its eventual decomposition, we do not have access to diffraction measurements made on cooling, and hence do not have a measure of the thermal hysteresis that must accompany this transition.

**Figure 5:**
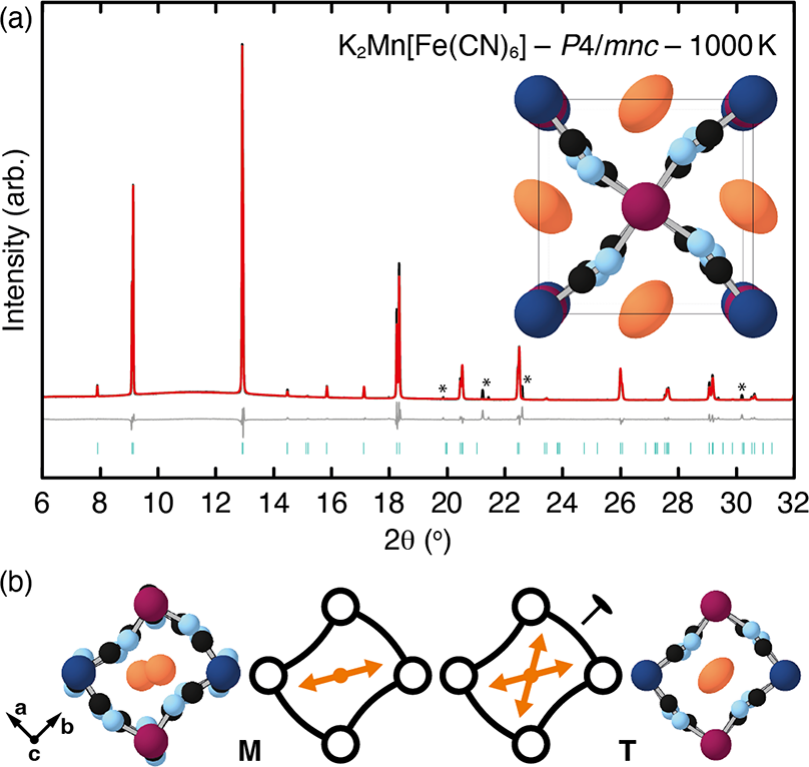
(a) Synchrotron X-ray powder diffraction pattern (λ = 0.825318(3) Å) and corresponding Rietveld fit for K_2_Mn[Fe(CN)_6_], measured at 1000 K. Data are shown in black, fit is shown in red, difference curve is shown in gray (data-fit) offset below the data, and reflection positions are shown as teal tick marks. Peaks due to a small impurity phase are marked with asterisks. The inset shows a representation of the structure of tetragonal K_2_Mn[Fe(CN)_6_], with anisotropic displacement ellipsoids for the K^+^ ions shown at 80% probability. (b) Within the monoclinic phase (“M”), K-ion displacements are predominantly along a ⟨110⟩ direction, shown here schematically as the almost-horizontal orange arrows. On transition to the higher-symmetry tetragonal phase (“T”) an additional diad operation (180° rotation) acting on the A-site superimposes symmetry-related displacement directions. The resulting distribution of equivalent sites is described by an anisotropic displacement ellipsoid elongated along the ⟨100⟩ direction of the parent structure.

By 1000 K the diffraction pattern is well accounted for by a structural model in which the K-ion slides are no longer present, but the primary tilt system remains active. This model has tetragonal *P*4/*mnc* space-group symmetry, as identified in our symmetry discussion above and as anticipated in the group-theoretical analysis of ref (39). The Rietveld fit obtained at 1000 K using this *P*4/*mnc* model is shown in Figure 5(a). A representation of the *P*4/*mnc* structural model is also included in Figure 5(a), from which it is clear that the *M*_3_^+^ tilt distortion remains strong at this temperature. An interesting feature of this structure is that the crystallographic point symmetry at the K^+^ site (Wyckoff position 4*d*) is 222, with principal axes aligned along the ⟨100⟩ directions of the parent cubic structure. Applying this point symmetry to the K-ion positions of the low-temperature monoclinic structure acts to average out the effects of the dominant ⟨110⟩ displacements but preserves in the orientation of the anisotropic displacement parameters the weaker contributions along ⟨100⟩. This is why the dominant displacement direction (now thermally averaged) shifts within this high-temperature phase from being toward an edge of the cube cavity to being toward a face of the cube instead [Figure 5(b)]. The particular ⟨100⟩ direction chosen is closely coupled to the tilt distortion and alternates in a herringbone fashion accordingly. Of course diffraction measurements are not sensitive to whether the displacement patterns are dominated by static or dynamic disorder.

**Figure 6:**
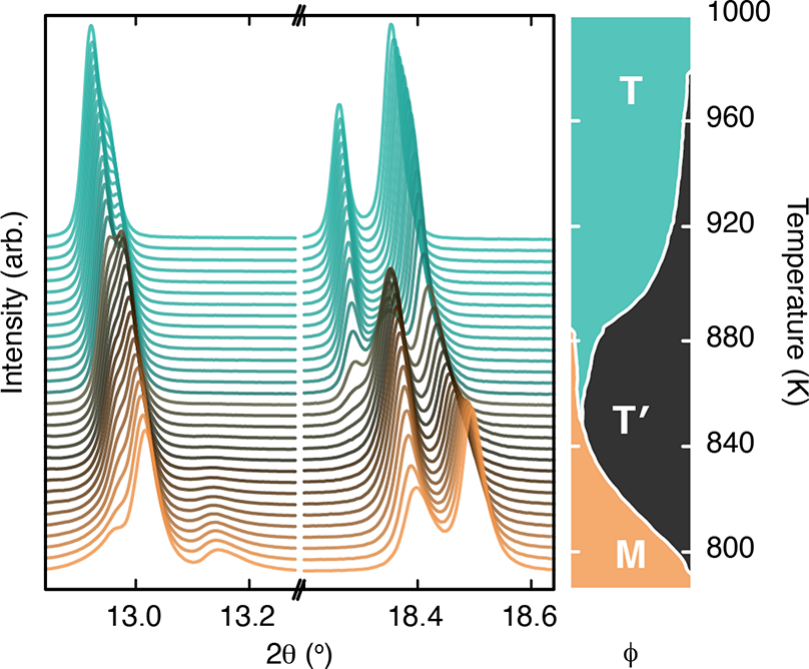
Section of synchrotron X-ray diffraction traces for K_2_Mn[Fe(CN)_6_ measured over the temperature range 800–1000 K. Successive curves are shifted vertically by a constant amount to show the evolution of the three phases. The color of each trace corresponds to temperature as shown in the right-hand plot, which includes the relative phase fraction ϕ of each PBA phase as a function of temperature.

The transition between ambient-temperature monoclinic and high-temperature tetragonal phases (hereafter denoted by the labels “M” and “T”, respectively) is not as straightforward as it might be. At intermediate temperatures 795 < *T* < 980 K we observe a transient intermediate phase that also appears to have tetragonal *P*4/*mnc* symmetry. We denote this phase by the label “T′” and show in Figure 6 the diffraction evidence for its existence. The first-order transition from M to T′ can be followed by the disappearance of satellite peaks such as that near 2θ = 13.2°. The transition from T′ to T is again first-order (as would be required by an isosymmetric transition^54^) and can be tracked via the sudden jump of peak positions as seen near 2θ = 18.4°. Given the close similarity of the three phases—both chemically and crystallographically—extreme caution was exercised when multiphase Rietveld refinements were carried out across the T′ (meta)stability regime. We leave discussion of the details to a relevant section in the Supporting Information, but note that the tetragonal distortion, degree of *M*_3_^+^ tilts, and form of the K-ion anisotropic displacement parameters differ between T′ and T phases.

### Generalized Phase Behavior

**Figure 7:**
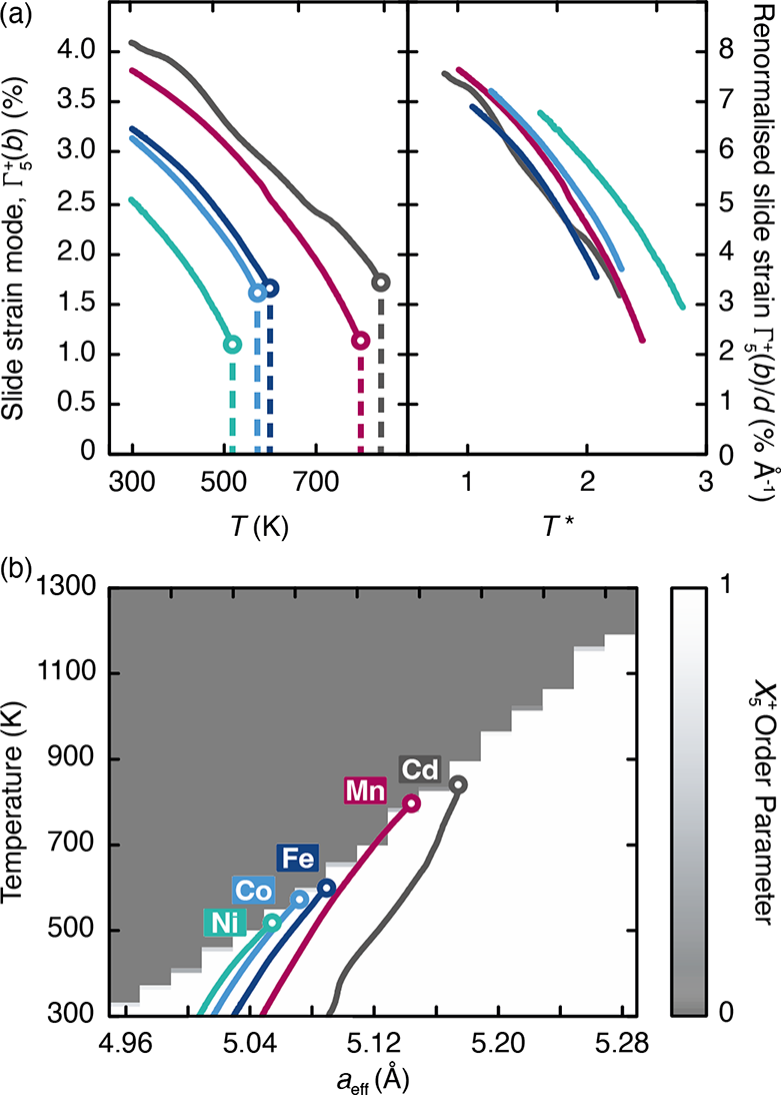
(a) Evolution of the monoclinic strain in K_2_M[Fe(CN)_6_] samples as a function of the composition and temperature. Colors denote M as in Figure 3(b), and the circles represent the limiting data at the point of transition to the high-temperature tetragonal phase (distortion amplitude identically zero). Data in the left-hand panel are shown in absolute units, and those on the right are shown in renormalized form as described in the text. (b) Degree of *X*_5_^+^ slide order as determined using Monte Carlo simulations, enumerated as a function of simulation temperature and effective lattice parameter. Superimposed in the foreground are traces of the experimental effective unit-cell constants for five K_2_M[Fe(CN)_6_] as a function of temperature, with transition temperatures marked with open circles as in (a). The experimental data in parts (a) and (b) are derived from the same series of variable-temperature synchrotron X-ray powder diffraction measurements.

Variable-temperature synchrotron X-ray powder diffraction measurements for all other K_2_M[Fe(CN)_6_] samples show analogous behavior across the entire family to that we have described for M = Mn. A summary of the key results is shown in the left-hand panel of Figure 7(a): in each case, the K-ion slide distortion is gradually removed with increasing temperature until a discontinuous transition at a critical temperature to a high-temperature tetragonal phase of *P*4/*mnc* symmetry. (The structural changes in K_2_Ni[Fe] were most challenging to track as a result of relatively strong strain effects that complicated multiphase refinement. We note also that K_2_Cd[Fe] showed partial decomposition on heating. A third comment is that the high-temperature K_2_Fe[Fe(CN)_6_] phase identified in ref (39) is probably also best interpreted in terms of this same *P*4/*mnc* model.) Varying composition affects at once both the magnitude of the K-ion slides (as we have already seen) and also the value of the phase-transition temperature. The value of *T*_c_ is lowest for M^2+^ with the smallest ionic radii. That the phase behavior of this family reflects a single common mechanism is a point made clear by renormalizing our experimental results to account for the effect of variation in unit-cell dimensions. For example, the absolute magnitude of the Γ_5_^+^(*b*) distortion will be larger for systems with greater *a*_eff_ (as established in eq 4), but we can track the relative distortion magnitude by dividing this parameter by the room-temperature K-ion displacement *d*. Likewise, increasing *a*_eff_ varies the dipolar coupling strength *D* according to eq 3, but again we can account for this change in energy scale by calculating the relative temperature T* = *T*/*D*. Recast in this way, our experimental results collapse onto a common trendline [right-hand panel of Figure 7(a)]. Even the data for M = Ni, which were the most difficult to obtain as a result of strain broadening, follow relatively closely this same universal behavior.

This (perhaps surprising) universality of behavior suggests that the extent, activation, and quenching of K-ion slide distortions in PBAs are essentially determined by geometry: the different chemistries of the various transition metals in our different samples have no obvious effect on PBA phase behavior beyond the influence of varying ionic radius alone. As a further test of this interpretation, we sought to compare our experimental observations with the predictions of the simple toy model of eq 2, a model that is based on geometric considerations alone.

In this spirit, we carried out a series of Monte Carlo (MC) simulations driven by eq 2 across a physically relevant range of *a*_eff_ values and temperatures. For a given effective unit-cell constant *a*_eff_, we first estimated the corresponding K-ion displacement magnitude *d* using eq 4. [For these simulations we employed a proportionality constant  (since K-ion displacements are along ⟨110⟩ directions), and an accordingly modified critical cell constant of *a*_*c*_ = 4.67 Å. The calculated phase behavior is remarkably resilient to variation in these parameters.] From this combination of *d* and *a*_eff_ the dipole–dipole coupling strength *D* is straightforwardly obtained [eq 3]. We expect the strain coupling *J* to vary as
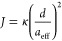
5where κ is a simple proportionality constant related to the elastic moduli. We make the assumption that variation in elastic constants will be small across our PBA series and so used a single common estimate κ ≃ *q*^2^/4*πϵ*_0_*a*_c_, chosen to bring *J* and *D* onto similar energy scales. Having determined the interaction energies *J* and *D* for a given *a*_eff_, we then carried out a series of MC simulations, starting at high temperatures (1300 K) and successively cooling to room temperature. After establishing equilibrium at a given temperature point, we calculated an *X*_5_^+^ order parameter from the MC configurations [cf. Figure 2]. The evolution of this order parameter as a function of *a*_eff_ and *T* is shown graphically in Figure 7(b). What is clear is that our simple toy model predicts increasing stabilization of K-ion slides as the effective unit-cell constant increases.

Figure 7(b) superimposes on these simulation results the experimental trajectories measured crystallographically for each of our K_2_M[Fe(CN)_6_] samples. The corresponding traces show positive slopes as a consequence of thermal expansion, the rate of which is similar across the series (see Supporting Information for further discussion). The experimentally determined phase-transition temperatures are marked as open circles, and we find remarkable agreement between the location of these values of *T*_c_ and the predictions of our simple MC simulations. It turns out that thermal expansion plays a significant role in stabilizing K-ion slides. In its absence, i.e., were the trajectories of Figure 7(b) given by vertical lines—then one would anticipate a reduction of *T*_c_ by several hundreds of Kelvin. This discrepancy explains why *T*_c_ is only ∼40% larger than *D* in MC simulations [Figure 2], but, experimentally, we observe K-ion slides to persist to *T*/*D* ≃ 2.5 [Figure 7(a)].

## Concluding Remarks

So the picture that emerges is as follows. The choice of M^2+^ cation in K_2_M[Fe(CN)_6_] PBAs determines their effective unit-cell dimensions through simple ionic radius considerations. The larger the cell, the greater the tendency for K-ions to displace away from the high-symmetry A-site. This displacement results in both a local electrostatic dipole and an elastic distortion of the surrounding cyanide framework as its symmetry is broken. Coupling between these two components from A-site to A-site leads to the cooperative K-ion slide distortion observed experimentally; this particular pattern of displacements can be rationalized on symmetry arguments alone. On heating, there is competition between thermal fluctuations of the K-ion positions, which act to quench the slides, and thermal expansion of the PBA framework, which enhances the tendency to off-center and hence stabilizes the slide distortion. The former is the stronger effect, and so the K-ion slides disorder at elevated temperatures, resulting in a phase transition to a higher-symmetry tetragonal structure. The critical temperature at which this transition occurs is higher for PBAs with larger unit cells, for which the dipole and strain interactions are stronger.

The microscopic model on which our calculations are based is intentionally simplistic, and one expects that in practice the thermal dependence of slide distortions is not purely order/disorder in character but rather includes a displacive component from variations in the effective K-ion potential well. This effect likely rationalizes the more gradual order parameter evolution observed experimentally for *T* < *T*_c_ than emerges from our model. Detailed characterization using a much higher level of theory (e.g., density functional theory) would provide important quantitative insight into the various distortion mechanisms at play, their interdependence, and the underlying energetics.^16,55^ The main advantage of a toy model, such as we employ here, is that it allows us to identify the central role of dipolar interactions in driving the K-ion slides and the primary effects of varying the composition and temperature. One further implication is that the same model may be relevant to a range of chemically different systems. Even among PBAs, for example, it is understood that Li^+^ and Na^+^ ions displace in different directions to K^+^, and hence the cooperative organization of these ions may be described by a variation of this same model. Likewise, our framework may be relevant too to studying orientational order/disorder processes of hybrid perovskites containing intrinsically polar A-site cations, such as methylammonium, which also polarizes along ⟨110⟩ in some lead halide frameworks at low temperatures.^49,56^ Drawing inspiration from compositional–property relationships in conventional perovskites,^57^ we anticipate the possibility of exploiting “slide engineering” through A-site compositional variation as an attractive avenue for functional materials design.

Returning the specific context of K-ion PBAs, the clear relationship between thermal and electrochemical (de)activation of K-ion slides identified in ref (39) suggests that the various trends we study here are likely to have ramifications for K-ion transport in PBAs more generally. For example, we infer that the unit-cell dimension varies the shape of the effective potential in which K^+^ ions sit, which in turn will affect the kinetic barriers to K-ion migration through the PBA framework. Understanding the interplay among PBA composition, K-ion occupancy, temperature, and K-ion diffusion kinetics would be a particularly interesting avenue for further investigation, with the potential benefit of identifying materials design strategies to optimize PBA cathode performance.

## Methods

### Synthesis

We synthesized samples of K_2_M[Fe(CN)_6_] (M = Ni, Co, Fe, Mn, Cd) in a similar fashion to ref (39) via a citrate-assisted precipitation in aqueous media. MSO_4_ (Sigma-Aldrich, 1 mmol) was dissolved in an aqueous solution of potassium citrate (Sigma-Aldrich, 0.5 M, 20 mL). This solution was added dropwise to a stoichiometric aqueous solution of K_4_Fe(CN)_6_ (Sigma-Aldrich, 20 mL) at 80 °C with stirring. The mixture was stirred for 2 h and then allowed to age for a further 2 h. The precipitate was isolated by centrifugation and washed with a 50:50 water/ethanol mixture in order to prevent the solid from dispersing. The solid was dried in air at 70 °C overnight.

### Materials Characterization

Synchrotron X-ray diffraction (XRD) measurements were performed on the I11 beamline of the Diamond Light Source operating with an X-ray wavelength of 0.825318(3) Å. The position-sensitive detector was used to collect diffraction patterns over the temperature range 300–1000 K with a hot-air blower. All Rietveld refinements were carried out using the TOPAS-Academic software.^52^

### Monte Carlo Simulations

Pseudospin Monte Carlo simulations were carried out using the code developed for ref (58), modified to allow the use of 12-state Potts degrees of freedom . This code makes use of Ewald summation to calculate long-range dipolar interactions, taking the numerical approach reported in ref (59). Simulations employed 8 × 8 × 8 supercells of the aristotypic *Pm*3̅*m* structure (i*.*e*. N* = 512 pseudospins). MC moves corresponded to random jumps between different Potts states for randomly selected pseudospins and were accepted or rejected according to the usual Metropolis criterion.^60^ Simulations were initialized using random pseudospin states, and simulated annealing runs were carried out from 1300 to 300 K, ensuring equilibration at each temperature step (Δ*T* = 40 K). All statistics reported were calculated over a series of independent runs (*N*_runs_ = 5). The *X*_5_^+^ order parameter was calculated as
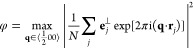
6where **r**_*j*_ denotes the position of pseudospin *j* and **e**_*j*_^⊥^ its orientation, projected onto a plane perpendicular to **q**.

## Supporting Information Available

Discussion of group theory, SEM, ICP-MS, XRD method, XRD refinement strategy, further XRD refinement analysis (PDF)
Tabulated crystallographic parameters obtained from Rietveld refinements described in the text (XLSX)

## References

[ref1] Kaye S. S.; Long J. R. Hydrogen storage in the dehydrated prussian blue analogues M_3_[Co(CN)_6_]_2_ (M = Mn, Fe, Co, Ni, Cu, Zn). J. Am. Chem. Soc. 2005, 127, 6506–6507. 10.1021/ja051168t. 15869251

[ref2] Pintado S.; Goberna-Ferrón S.; Escudero-Adán E. C.; Galán-Mascarós J. R. Fast and persistent electrocatalytic water oxidation by Co-Fe Prussian blue coordination polymers. J. Am. Chem. Soc. 2013, 135, 13270–13273. 10.1021/ja406242y. 23978044

[ref3] Bleuzen A.; Lomenech C.; Escax V.; Villain F.; Varret F.; Cartier Dit Moulin C.; Verdaguer M. Photoinduced Ferrimagnetic Systems in Prussian Blue Analogues C_*x*_^I^Co_4_[Fe(CN)_6_]_*y*_ (C^I^ = Alkali Cation). 1. Conditions to Observe the Phenomenon. J. Am. Chem. Soc. 2000, 122, 6648–6652. 10.1021/ja000348u.

[ref4] Ferlay S.; Mallah T.; Ouahès R.; Veillet P.; Verdaguer M. A room-temperature organometallic magnet based on prussian blue. Nature 1995, 378, 701–703. 10.1038/378701a0.

[ref5] Pasta M.; Wessells C. D.; Liu N.; Nelson J.; McDowell M. T.; Huggins R. A.; Toney M. F.; Cui Y. Full open-framework batteries for stationary energy storage. Nat. Commun. 2014, 5, 3007 10.1038/ncomms4007. 24389854

[ref6] Li W.; Wang Z.; Deschler F.; Gao S.; Friend R. H.; Cheetham A. K. Chemically diverse and multifunctional hybrid organic-inorganic perovskites. Nat. Rev. Mater. 2017, 2, 16099 10.1038/natrevmats.2016.99.

[ref7] Boström H. L.; Goodwin A. L. Hybrid Perovskites, Metal-Organic Frameworks, and Beyond: Unconventional Degrees of Freedom in Molecular Frameworks. Acc. Chem. Res. 2021, 54, 1288–1297. 10.1021/acs.accounts.0c00797. 33600147 PMC7931445

[ref8] Cattermull J.; Pasta M.; Goodwin A. L. Structural complexity in Prussian blue analogues. Mater. Horiz. 2021, 8, 3178–3186. 10.1039/D1MH01124C. 34713885 PMC9326455

[ref9] Goodwin A. L.; Chapman K. W.; Kepert C. J. Guest-dependent negative thermal expansion in nanoporous prussian blue analogues M^II^Pt^IV^(CN)_6_; M = Zn, Cd). J. Am. Chem. Soc. 2005, 127, 17980–17981. 10.1021/ja056460f. 16366530

[ref10] Chapman K. W.; Chupas P. J.; Kepert C. J. Compositional dependence of negative thermal expansion in the Prussian blue analogues M^II^Pt^IV^(CN)_6_ (M = Mn, Fe, Co, Ni, Cu, Zn, Cd). J. Am. Chem. Soc. 2006, 128, 7009–7014. 10.1021/ja060916r. 16719481

[ref11] Moritomo Y.; Kurihara Y.; Matsuda T.; Kim J. Structural phase diagram of Mn-Fe cyanide against cation concentration. J. Phys. Soc. Jpn. 2011, 80, 103601 10.1143/JPSJ.80.103601.

[ref12] Moritomo Y.; Matsuda T.; Kurihara Y.; Kim J. Cubic-rhombohedral structural phase transition in Na_1.32_Mn[Fe(CN)_6_]_0.83_ ·3.6H_2_O. J. Phys. Soc. Jpn. 2011, 80, 074608 10.1143/JPSJ.80.074608.

[ref13] Boström H. L. B.; Collings I. E.; Cairns A. B.; Romao C. P.; Goodwin A. L. High-pressure behaviour of Prussian blue analogues: Interplay of hydration, Jahn-Teller distortions and vacancies. Dalt. Trans. 2019, 48, 1647–1655. 10.1039/C8DT04463E. 30548036

[ref14] Benedek N. A.; Fennie C. J. Hybrid improper ferroelectricity: A mechanism for controllable polarization-magnetization coupling. Phys. Rev. Lett. 2011, 106, 3–6. 10.1103/PhysRevLett.106.107204. 21469829

[ref15] Rondinelli J. M.; Fennie C. J. Octahedral rotation-induced ferroelectricity in cation ordered perovskites. Adv. Mater. 2012, 24, 1961–1968. 10.1002/adma.201104674. 22488734

[ref16] Benedek N. A.; Rondinelli J. M.; Djani H.; Ghosez P.; Lightfoot P. Understanding ferroelectricity in layered perovskites: New ideas and insights from theory and experiments. Dalt. Trans. 2015, 44, 10543–10558. 10.1039/C5DT00010F. 25687622

[ref17] Senn M. S.; Bombardi A.; Murray C. A.; Vecchini C.; Scherillo A.; Luo X.; Cheong S. W. Negative thermal expansion in hybrid improper ferroelectric Ruddlesden-popper Perovskites by symmetry trapping. Phys. Rev. Lett. 2015, 114, 23–27. 10.1103/PhysRevLett.114.035701. 25659007

[ref18] Boström H. L.; Senn M. S.; Goodwin A. L. Recipes for improper ferroelectricity in molecular perovskites. Nat. Commun. 2018, 9, 2380 10.1038/s41467-018-04764-x. 29915202 PMC6006342

[ref19] Pitcher M. J.; Mandal P.; Dyer M. S.; Alaria J.; Borisov P.; Niu H.; Claridge J. B.; Rosseinsky M. J. Tilt engineering of spontaneous polarization and magnetization above 300 K in a bulk layered perovskite. Science 2015, 347, 420–424. 10.1126/science.1262118. 25613888

[ref20] Simonov A.; De Baerdemaeker T.; Boström H. L. B.; Ríos Gómez M. L.; Gray H. J.; Chernyshov D.; Bosak A.; Bürgi H. B.; Goodwin A. L. Hidden diversity of vacancy networks in Prussian blue analogues. Nature 2020, 578, 256–260. 10.1038/s41586-020-1980-y. 32051599 PMC7025896

[ref21] Boström H. L. B.; Brant W. R. Octahedral tilting in Prussian blue analogues. J. Mater. Chem. C 2022, 10, 13690–13699. 10.1039/D2TC00848C.

[ref22] Nielsen I.; Dzodan D.; Ojwang D. O.; Henry P. F.; Ulander A.; Ek G.; Häggström L.; Ericsson T.; Boström H. L.; Brant W. R. Water driven phase transitions in Prussian white cathode materials. J. Phys. Energy 2022, 4, 044012 10.1088/2515-7655/ac9808.

[ref23] Peng J.; Huang J.; Gao Y.; Qiao Y.; Dong H.; Liu Y.; Li L.; Wang J.; Dou S.; Chou S. Defect-Healing Induced Monoclinic Iron-Based Prussian Blue Analogs as High-Performance Cathode Materials for Sodium-Ion Batteries.. Small 2023, 19, 2300435 10.1002/smll.202300435. 37166020

[ref24] Dhir S.; Wheeler S.; Capone I.; Pasta M. Outlook on K-Ion Batteries. Chem. 2020, 6, 2442–2460. 10.1016/j.chempr.2020.08.012.

[ref25] Wu X.; Wu C.; Wei C.; Hu L.; Qian J.; Cao Y.; Ai X.; Wang J.; Yang H. Highly Crystallized Na_2_CoFe(CN)_6_ with Suppressed Lattice Defects as Superior Cathode Material for Sodium-Ion Batteries. ACS Appl. Mater. Interfaces 2016, 8, 5393–5399. 10.1021/acsami.5b12620. 26849278

[ref26] Bie X.; Kubota K.; Hosaka T.; Chihara K.; Komaba S. A novel K-ion battery: hexacyanoferrate(II)/graphite cell. J. Mater. Chem. A 2017, 5, 4325–4330. 10.1039/C7TA00220C.

[ref27] Hosaka T.; Fukabori T.; Kojima H.; Kubota K.; Komaba S. Effect of Particle Size and Anion Vacancy on Electrochemical Potassium Ion Insertion into Potassium Manganese Hexacyanoferrates. ChemSusChem 2021, 14, 1166–1175. 10.1002/cssc.202002628. 33369231

[ref28] Liu H.; Stonebridge F. C.; Borkiewicz O. J.; Wiaderek K. M.; Chapman K. W.; Chupas P. J.; Grey C. P. Capturing metastable structures during high-rate cycling of LiFePO_4_ nanoparticle electrodes. Science 2014, 344, 1252817 10.1126/science.1252817. 24970091

[ref29] Ohno S.; Banik A.; Dewald G. F.; Kraft M. A.; Krauskopf T.; Minafra N.; Till P.; Weiss M.; Zeier W. G. Materials design of ionic conductors for solid state batteries. Prog. Energy 2020, 2, 022001 10.1088/2516-1083/ab73dd.

[ref30] Cattermull J.; Pasta M.; Goodwin A. L. Predicting Distortion Magnitudes in Prussian Blue Analogues. ChemRxiv 2023, 10.26434/chemrxiv–2023–f34l6. PMC10655185 37931061

[ref31] Goodwin A. L. Rigid unit modes and intrinsic flexibility in linearly bridged framework structures. Phys. Rev. B 2006, 74, 124302 10.1103/PhysRevB.74.134302.

[ref32] Glazer A. M. The classification of tilted octahedra in perovskites. Acta Crystallogr. 1972, B28, 3384–3392. 10.1107/S0567740872007976.

[ref33] Howard C. J.; Stokes H. T. Group-Theoretical Analysis of Octahedral Tilting in Perovskites. Acta Crystallogr. 1998, B54, 782–789. 10.1107/S0108768198004200. 12456971

[ref34] Goodenough J. B. Theory of the role of covalence in the perovskite-type manganites [La,M(II)]MnO_3_. Phys. Rev. 1955, 100, 564–573. 10.1103/PhysRev.100.564.

[ref35] Goodenough J. B. Jahn-Teller phenomena in solids. Annu. Rev. Mater. Sci. 1998, 28, 1–27. 10.1146/annurev.matsci.28.1.1.

[ref36] Howard C. J.; Carpenter M. A. Octahedral tilting in cation-ordered Jahn-Teller distorted perovskites - A group-theoretical analysis. Acta Crystallogr. 2010, B66, 40–50. 10.1107/S0108768109048010. 20101082

[ref37] Duyker S. G.; Hill J. A.; Howard C. J.; Goodwin A. L. Guest-Activated Forbidden Tilts in a Molecular Perovskite Analogue. J. Am. Chem. Soc. 2016, 138, 11121–11123. 10.1021/jacs.6b06785. 27533044

[ref38] Boström H. L.; Hill J. A.; Goodwin A. L. Columnar shifts as symmetry-breaking degrees of freedom in molecular perovskites. Phys. Chem. Chem. Phys. 2016, 18, 31881–31894. 10.1039/C6CP05730F. 27841402

[ref39] Cattermull J.; Sada K.; Hurlbutt K.; Cassidy S. J.; Pasta M.; Goodwin A. L. Uncovering the Interplay of Competing Distortions in the Prussian Blue Analogue K_2_Cu[Fe(CN)_6_]. Chem. Mater. 2022, 34, 5000–5008. 10.1021/acs.chemmater.2c00288. 35722203 PMC9202302

[ref40] Burdett J. K. Use of the Jahn-Teller Theorem in Inorganic Chemistry. Inorg. Chem. 1981, 20, 1959–1962. 10.1021/ic50221a003.

[ref41] Cohen R. E. Origin of ferroelectricity in perovskite oxides. Nature 1992, 359, 136–138. 10.1038/358136a0.

[ref42] Bradley C.; Cracknell A. Math. Theory Symmetry Solids; Oxford Classic Texts in the Physical Sciences; Clarendon Press: Oxford, 1972; pp 15–24.

[ref43] Woodward P. M. Octahedral Tilting in Perovskites. I. Geometrical Considerations. Acta Crystallogr. 1997, B53, 32–43. 10.1107/S0108768196010713.

[ref44] Stokes H. T.; Hatch D. M.; Campbell B. J. ISODISTORT.; Brigham Young University: Provo, UT, 2022.

[ref45] Campbell B. J.; Stokes H. T.; Tanner D. E.; Hatch D. M. ISODISPLACE: A web-based tool for exploring structural distortions. J. Appl. Crystallogr. 2006, 39, 607–614. 10.1107/S0021889806014075.

[ref46] Senn M. S.; Bristowe N. C. A group-theoretical approach to enumerating magnetoelectric and multiferroic couplings in perovskites. Acta Crystallogr. 2018, A74, 308–321. 10.1107/S2053273318007441. PMC6038361 29978842

[ref47] Paddison J. A. M.; Jacobsen H.; Petrenko O. A.; Fernández-Díaz M. T.; Deen P. P.; Goodwin A. L. Hidden order in spin-liquid Gd_3_Ga_5_O_12_. Science 2015, 350, 179–181. 10.1126/science.aaa5326. 26450205

[ref48] Belobrov P. I.; Gekht R. S.; Ignatchenko V. A. Ground-state in systems with dipole interactions. Zh. Eksp. Teor. Friz. 1983, 84, 1097–1108.

[ref49] Leguy A. M.; Frost J. M.; McMahon A. P.; Sakai V. G.; Kochelmann W.; Law C.; Li X.; Foglia F.; Walsh A.; O’Regan B. C.; Nelson J.; Cabral J. T.; Barnes P. R. The dynamics of methylammonium ions in hybrid organic-inorganic perovskite solar cells. Nat. Commun. 2015, 6, 7124 10.1038/ncomms8124. 26023041 PMC4458867

[ref50] Coates C. S.; Gray H. J.; Bulled J. M.; Boström H. L. B.; Simonov A.; Goodwin A. L. Ferroic multipolar order and disorder in cyanoelpasolite molecular perovskites. Philos. Trans. R. Soc. A Math. Phys. Eng. Sci. 2019, 377, 20180219 10.1098/rsta.2018.0219. PMC6562344 31130093

[ref51] Allen D. J.; Bristowe N. C.; Goodwin A. L.; Yeung H. H. Mechanisms for collective inversion-symmetry breaking in dabconium perovskite ferroelectrics. J. Mater. Chem. C 2021, 9, 2706–2711. 10.1039/D1TC00619C. PMC8905487 35359799

[ref52] Coelho A. A. TOPAS-Academic, version 6; Coelho Software: Brisbane, 2016.

[ref53] Shannon R. D. Revised Effective Ionic Radii and Systematic Studies of Interatomie Distances in Halides and Chaleogenides. Acta Crystallogr. 1976, A32, 751–767. 10.1107/S0567739476001551.

[ref54] Christy A. G. Isosymmetric structural phase transitions: phenomenology and examples. Acta Crystallogr. 1995, B51, 753–757. 10.1107/S0108768195001728.

[ref55] Lee J. H.; Bristowe N. C.; Lee J. H.; Lee S. H.; Bristowe P. D.; Cheetham A. K.; Jang H. M. Resolving the Physical Origin of Octahedral Tilting in Halide Perovskites. Chem. Mater. 2016, 28, 4259–4266. 10.1021/acs.chemmater.6b00968.

[ref56] Swainson I. P.; Hammond R. P.; Soullière C.; Knop O.; Massa W. Phase transitions in the perovskite methylammonium lead bromide, CH_3_ND_3_PbBr_3_. J. Solid State Chem. 2003, 176, 97–104. 10.1016/S0022-4596(03)00352-9.

[ref57] Attfield J. P. ‘A’ cation control of perovskite properties. Cryst. Eng. 2002, 5, 427–438. 10.1016/S1463-0184(02)00054-0.

[ref58] Paddison J. A. M.; Goodwin A. L. Empirical magnetic structure solution of frustrated spin systems. Phys. Rev. Lett. 2012, 108, 017204 10.1103/PhysRevLett.108.017204. 22304284

[ref59] Wang Z.; Holm C. Estimate of the cutoff errors in the Ewald summation for dipolar systems. J. Chem. Phys. 2001, 115, 6351–6359. 10.1063/1.1398588.

[ref60] Metropolis N.; Rosenbluth A. W.; Rosenbluth M. N.; Teller A. H.; Teller E. Equation of state calculations by fast computing machines. J. Chem. Phys. 1953, 21, 1087–1092. 10.1063/1.1699114. 41342507

