# Supplementary material for: K-Ion Slides in Prussian Blue Analogues
Source: J Am Chem Soc. 2023 Oct 25;145(44):24249–59. doi: 10.1021/jacs.3c08751 (PMC10636749; doi:10.1021/jacs.3c08751)
Supplement: Supplementary file 1 — ja3c08751_si_001.pdf [file ja3c08751_si_001.pdf]

K-ion slides in Prussian Blue Analogues

SUPPLEMENTARY INFORMATION

John Cattermull,[a,b] Nikolaj Roth,[a,c] Simon Cassidy,[a] Mauro Pasta[b] and and Andrew L. Goodwin[a*]

[a]Department of Chemistry, University of Oxford, Inorganic Chemistry Laboratory,
South Parks Road, Oxford OX1 3QR, U.K.

[b]Department of Materials, University of Oxford, Parks Road, Oxford OX1 3PH, U.K.

[c]iNANO, Aarhus, DK-8000 Denmark

*To whom correspondence should be addressed;
E-mail: andrew.goodwin@chem.ox.ac.uk

Contents

1 Group Theory 3

2 Scanning Electron Microscopy (SEM) 4

3 Elemental Analysis by Inductively Coupled Plasma Mass Spectrometry (ICP-MS) 5

4 Powder X-ray diffraction 6

5 References 20

1 Group Theory

Table S1: Distortion modes for and corresponding distortion magnitudes at room temperature for $K_2Mn[Fe(CN)_6]$ relative to the unit cell given in Table S3. Primary distortions as discussed in the main text are highlighted in blue; the remaining distortions are secondary. Note that some irrep labels differ from those discussed in the main text as the parent structure has $Fm\bar{3}m$ symmetry and incorporates B-site order.

Irrep	Atom/Lattice Parameter(s)	Magnitude	Comment
Γ_1^+	$+a, +b, +c$	−0.00239(14)	Isotropic volume strain
Γ_3^+	$+a, -b, -c$	−0.0001(2)	Tetragonal strain
$\Gamma_5^+(a)$	$-\beta$	−0.00014(1)	Monoclinic strain
$\Gamma_5^+(b)$	$-b, +c$	−0.03817(0)	Monoclinic strain
R_1^+	C	0.035(7)	
R_3^+	C	−0.001(10)	
R_4^+	C	1.410(10)	$a^0b^-b^-$ tilt
$R_5^+(a)$	C	0.0221(10)	
$R_5^+(b)$	C	0.110(8)	
M_2^+	C	−0.093(10)	
M_3^+	C	1.099(11)	$a^0a^0c^+$ tilt
$M_5^+(1)$	C	0.021(11)	
$M_5^+(2)$	C	0.186(11)	
$R_5^+(a)$	K	−0.294(16)	$\langle 100\rangle$ K-ion displacements
$R_5^+(b)$	K	0.021(5)	
X_5^+	K	0.9341(10)	$\langle 110\rangle$ K-ion displacements
R_1^+	N	0.098(6)	
R_3^+	N	0.031(9)	
R_4^+	N	2.138(7)	$a^0b^-b^-$ tilt
$R_5^+(a)$	N	0.067(8)	
$R_5^+(b)$	N	0.058(8)	
M_2^+	N	−0.071(7)	
M_3^+	N	1.574(8)	$a^0a^0c^+$ tilt
$M_5^+(1)$	N	0.096(9)	
$M_5^+(2)$	N	0.428(8)	

2 Scanning Electron Microscopy (SEM)

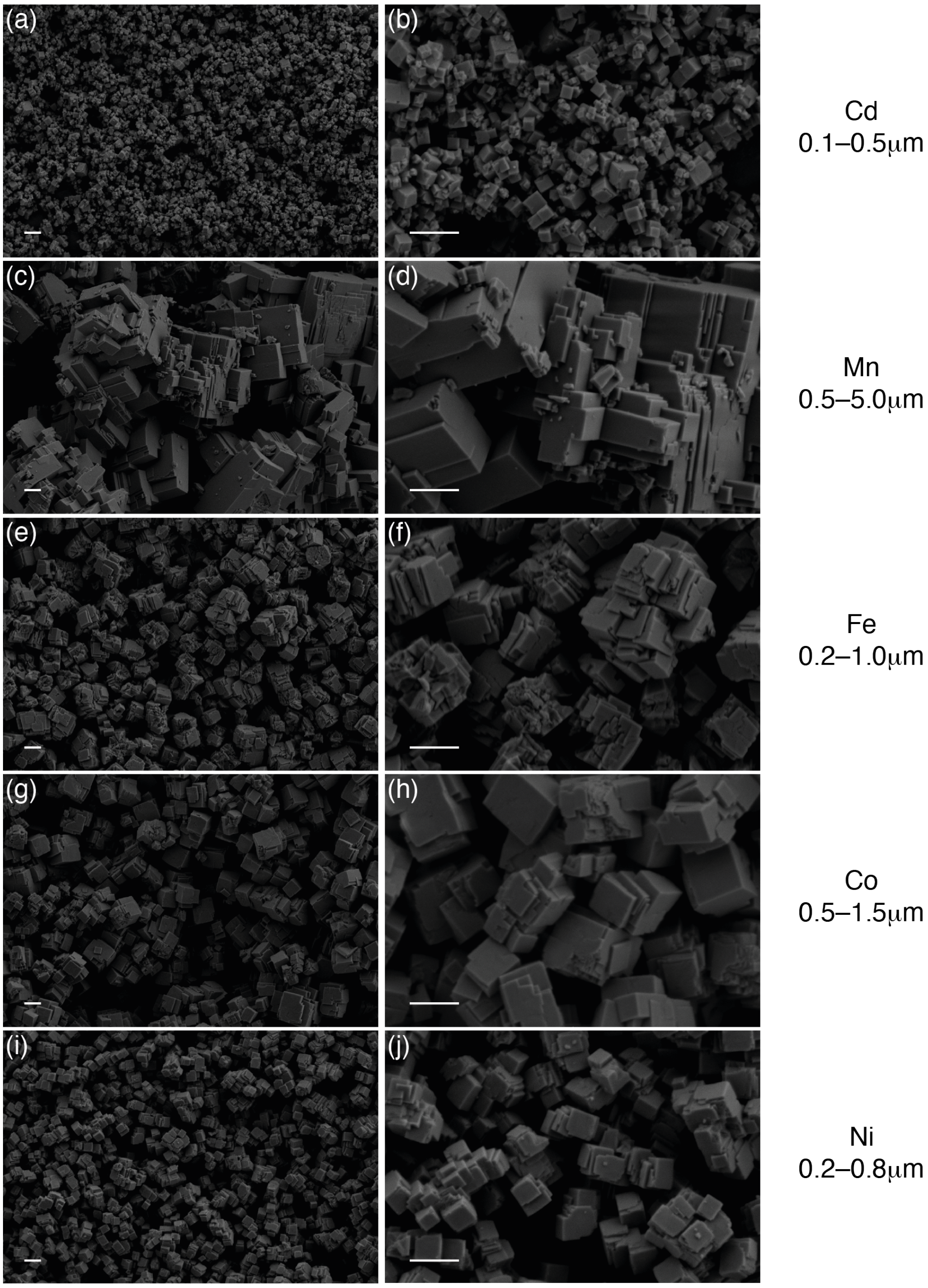

Figure S1: Scanning electron micrographs of samples $K_2M[Fe(CN)_6]$ with corresponding label for M and estimate of particle size. The variation in particle size scales somewhat with peak width in the XRD [Fig. 3(b)]. Each sample has corresponding two micrographs. The scale bar on each micrograph is 1 μ m. Domains in each crystal are clear from the aggregation of crystallites.

3 Elemental Analysis by Inductively Coupled Plasma Mass Spectrometry (ICP-MS)

All samples were tested for elemental composition by ICP-MS. The main use of this technique is to measure the ratio of the transition metal to estimate vacancy content, where K^+ concentration can be inferred by charge balancing. This technique is notoriously unreliable in determining potassium content,[S1] but it is supported by evidence from the Rietveld refinement of the room temperature XRD patterns.

Each $K_2M[Fe(CN)_6]$ sample was digested in concentrated HCl and then diluted in 2% nitric acid to give a final M concentration of approximately 70 parts per billion (ppb). Calibration for M, and Fe compositions was achieved through a series of standard nitrate solutions (Certipur, Merck) of known compositions; we used final concentrations of 0, 1, 2, 5, 10, 20, 40, 60, 80, 100 ppb. For our sample and for each standard solution, the corresponding M and Fe compositions were determined using a Shimadzu ICPMS-2030 spectrometer coupled with a mini torch and an AS-10 autosampler. A helium collision cell was used to remove interfering polyatomic species. Measurements were performed in triplicate, with washing runs carried out between successive measurements. The measured ICP-MS intensities for the $K_2M[Fe(CN)_6]$ samples are given in Table S2, together with the calculated M and Fe concentrations. The ratio of Fe/M gives an estimate of y in $K_xM[Fe(CN)_6]_y$. This could not be estimated for $K_2Fe[Fe(CN)_6]$ since both TM sites are indistinguishable by elemental analysis. The $K_2Cd[Fe(CN)_6]$ sample was particularly challenging to digest, making the reported ratio and vacancy content less reliable.

Table S2: Average ICP-MS data for each $K_2M[Fe(CN)_6]$ sample

M A	ρ(M)/μg dm^{-3}	c(M)/mol dm^{-3}	ρ(Fe 56)/μg dm^{-3}	c(Fe 56)/mol dm^{-3}	ratio
Cd 114	89.7(3)	0.787(3)	39.0(3)	0.696(4)	0.884(7)
Mn 55	56.2(3)	1.022(5)	57.1(3)	1.0(5)	0.98(5)
Fe 56	90.1(4)	1.610(6)	—	—	—
Co 59	58.5(10)	0.992(2)	56(3)	1.00(5)	1.01(5)
Ni 58	54.5(5)	0.940(8)	52.6(3)	0.939(5)	0.998(10)

4 Powder X-ray diffraction

Measurements

Our powder X-ray diffraction data made use of the synchrotron X-ray source on the I11 beamline at Diamond Light Source, UK. The diffraction patterns of the sample were collected in capillary transmission geometry. A room temperature X-ray diffraction pattern was collected using the Mythen2 Position Sensitive Detector (PSD), two data collections of 20 seconds each were taken at angles 0.25 degrees apart, then summed to account for gaps in the detector coverage.

Measurements between 300 and 1000 K were performed using an FMB Oxford cyberstar hot air blower aimed side-on to the sample at the beam position. Data were collected by continually warming the sample at a rate of 6 K min^{-1} while collecting two 5 second scans with the PSD.

Ambient-temperature data: refinement details

The structural refinement for room temperature $K_2M[Fe(CN)_6]$ M = Cd, Mn, Fe, Co, Ni) was performed using a Rietveld refinement in TOPAS software,[S2] in conjunction with distortion modes obtained from ISODISTORT.[S3, S4] The parent cell was a cubic crystal structure of $K_2M[Fe(CN)_6]$ in $Fm\bar{3}m$ taken from the undistorted cubic cell from Ref. S5, with crystallographic details given in Table S3. K occupancies were allowed to refine freely and refinements returned an occupancy of at least 1.95 for all samples. The uncertainties reported from the refinement in TOPAS are likely overly precise, hence why ICP-MS was employed to back-up the evidence. As further support for the sensitivity of the refinement to K occupancy a fit is included in Fig. S2 where the K occupancy was fixed to $K_{1.6}Mn[Fe(CN)_6]$.

Positions of all of the atoms were allowed to refine as a function of the 21 distortion modes generated by a distortion from $Fm\bar{3}m$ to $P2_1/n$, many of which were minor in contribution, but allowed by symmetry. In order to reduce the number of free parameters, the thermal displacement parameters for transition metals M and Fe were constrained to be equal; so too for those of the C and N sites. The K site was allowed to refine with an independent thermal displacement parameter. Finally, an anisotropic peakshape model for an monoclinic unit cell was applied to account for the different strains in different directions that individual crystallites will have, giving a better interpretation of the peak intensities.[S6]

Table S3: Crystallographic details for the $Fm\bar{3}m$ parent cubic structure.[S5]

a(Å)	10.11349		
Z	4		
Atom	x	y	z
C	0.1821	0.0	0.0
Fe	0.0	0.0	0.0
K	0.25	0.25	0.25
M	0.5	0.0	0.0
N	0.289	0.0	0.0

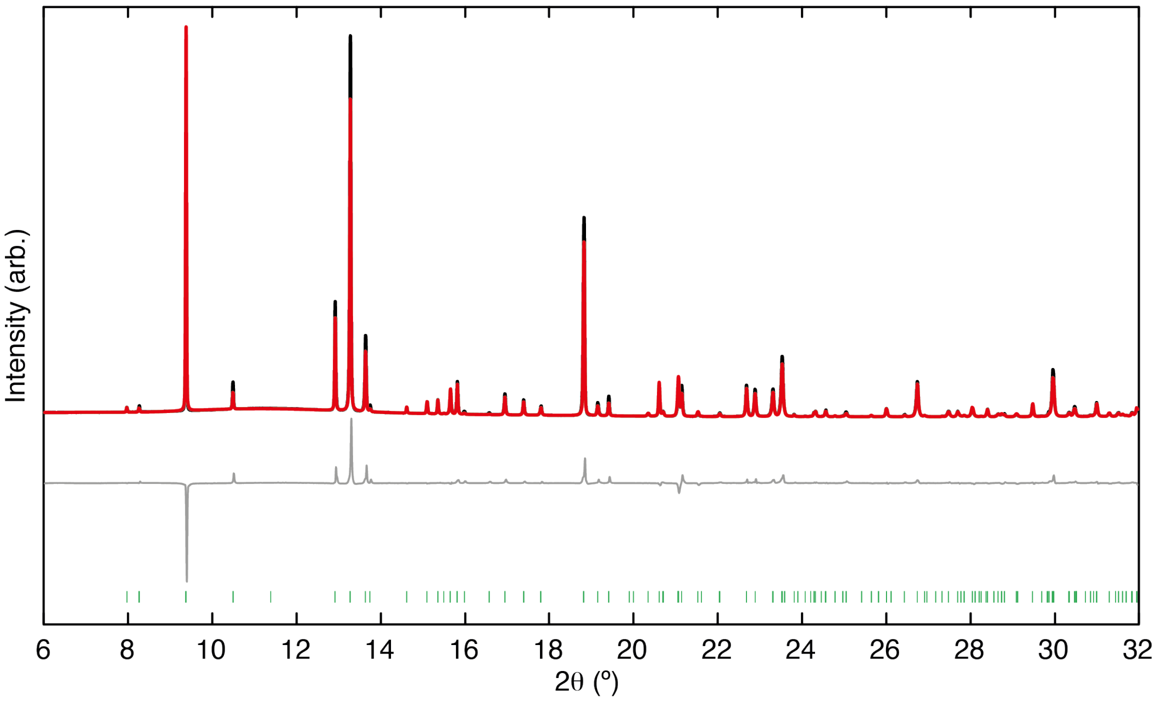

Figure S2: Rietveld fit to the X-ray powder diffraction pattern measured for $K_2Mn[Fe(CN)_6]$ at room temperature, with fixed K occupancy to give the formula $K_{1.6}Mn[Fe(CN)_6]$. Raw data in black, fit in red, and corresponding difference curve in grey (data – fit) is offset below the data. Tick marks denote reflection positions.

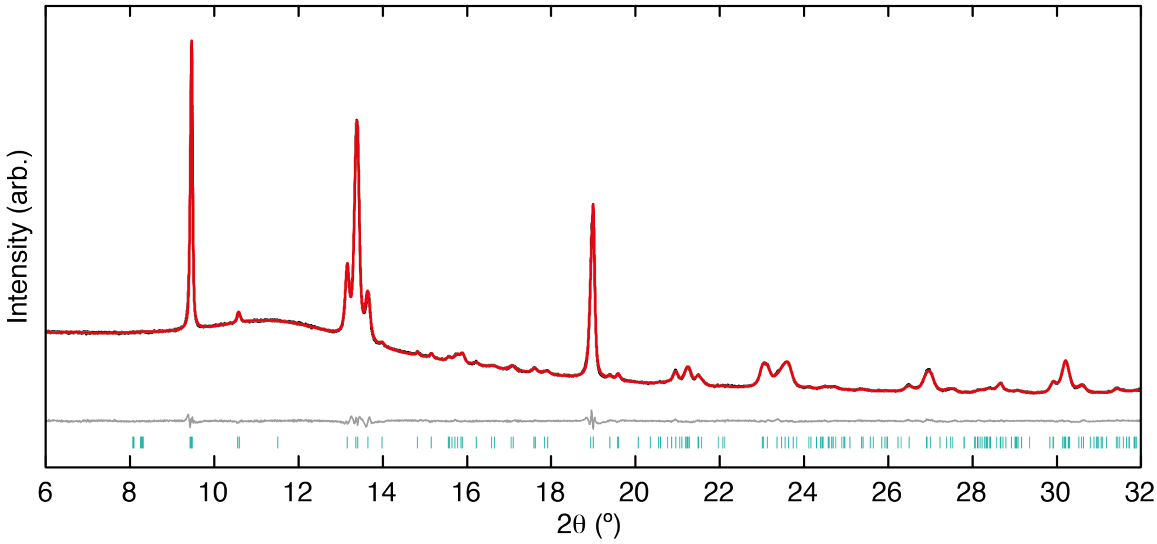

Figure S3: Rietveld fit to the X-ray powder diffraction pattern measured for $K_2Ni[Fe(CN)_6]$ at room temperature. Raw data in black, fit in red, and corresponding difference curve in grey (data – fit) is offset below the data. Tick marks denote reflection positions.

Table S4: Crystallographic parameters for $P2_1/n$ structure of $K_{1.986(2)}Ni[Fe(CN)_6]$ at room temperature from the fit the data in Fig. S3.

a (Å)	10.033(10)			
b (Å)	7.204(3)			
c (Å)	6.949(3)			
β (°)	89.717(3)			
V (Å^3)	502.2(6)			
Z	4			
Atom	x	y	z	U_{iso} (Å^2)
Fe	0	0	0	0.0077(4)
Ni	0.5	0	0	0.0077
K	0.7517(7)	0.54964(18)	−0.0149(3)	0.0434(6)
C1	−0.0282(13)	0.862(2)	0.777(2)	0.0101(10)
C2	−0.0169(13)	0.217(2)	0.858(2)	0.0101
C3	0.8092(14)	−0.005(2)	0.0401(17)	0.0101
N1	−0.0405(10)	0.7342(16)	0.6694(16)	0.0101
N2	0.9711(10)	0.3302(16)	0.7473(16)	0.0101
N3	0.7027(12)	−0.0163(17)	0.0883(11)	0.0101

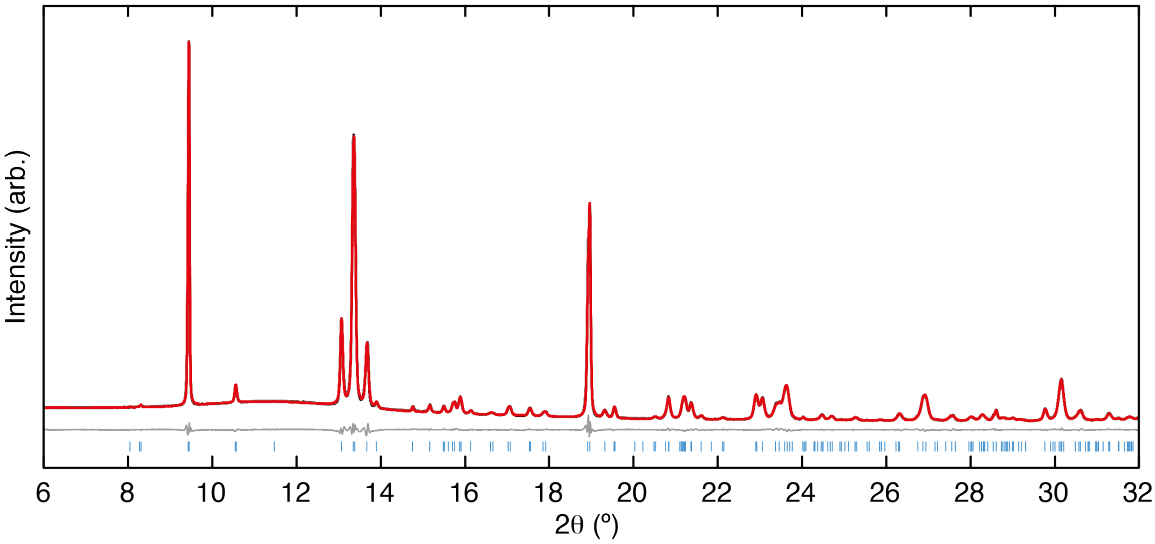

Figure S4: Rietveld fit to the X-ray powder diffraction pattern measured for $K_2Co[Fe(CN)_6]$ at room temperature. Raw data in black, fit in red, and corresponding difference curve in grey (data – fit) is offset below the data. Tick marks denote reflection positions.

Table S5: Crystallographic parameters for $P2_1/n$ structure of $K_{1.9708(16)}Co[Fe(CN)_6]$ at room temperature from the fit the data in Fig. S4.

a (Å)	10.045(17)			
b (Å)	7.251(5)			
c (Å)	6.933(5)			
β (°)	89.7804(11)			
V (Å^3)	505.0(10)			
Z	4			
Atom	x	y	z	U_{iso} (Å^2)
Fe	0	0	0	0.01016(14)
Co	0.5	0	0	0.01016
K	0.7506(2)	0.55759(7)	−0.01741(13)	0.0360(3)
C1	−0.0275(5)	0.8528(7)	0.7828(7)	0.0099(4)
C2	−0.0283(5)	0.2088(7)	0.8533(7)	0.0099
C3	0.8109(5)	−0.0016(7)	0.0569(6)	0.0099
N1	−0.0397(4)	0.7515(5)	0.6583(5)	0.0099
N2	0.9537(4)	0.3360(5)	0.7564(5)	0.0099
N3	0.7013(4)	−0.0265(6)	0.0867(5)	0.0099

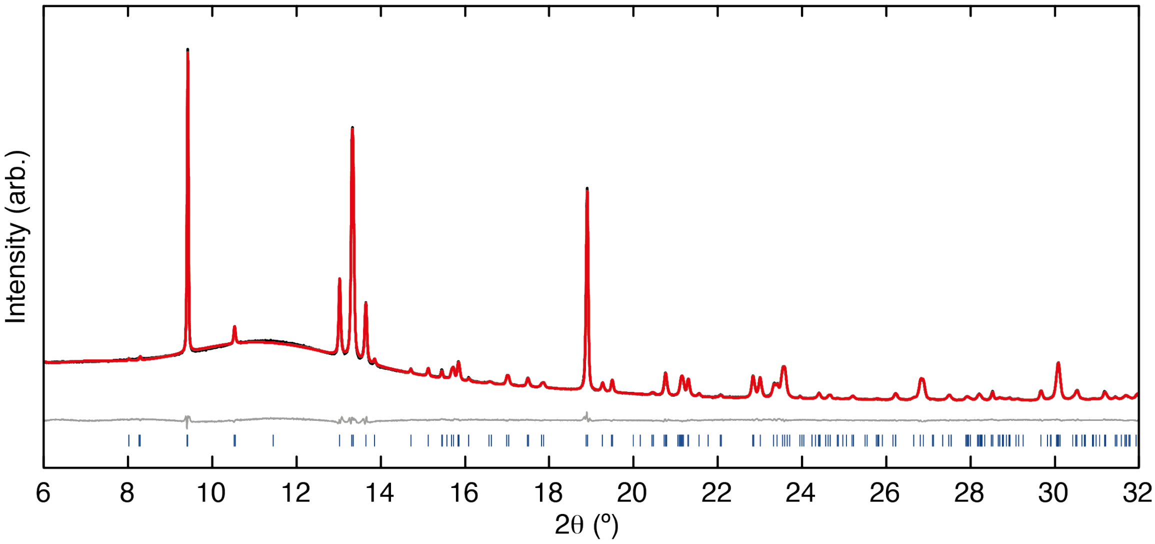

Figure S5: Rietveld fit to the X-ray powder diffraction pattern measured for $K_2Fe[Fe(CN)_6]$ at room temperature. Raw data in black, fit in red, and corresponding difference curve in grey (data – fit) is offset below the data. Tick marks denote reflection positions.

Table S6: Crystallographic parameters for $P2_1/n$ structure of $K_{1.946(2)}Fe[Fe(CN)_6]$ at room temperature from the fit the data in Fig. S5

a (Å)	10.063(19)			
b (Å)	7.276(5)			
c (Å)	6.948(5)			
β (°)	89.7977(7)			
V (Å^3)	508.8(12)			
Z	4			
Atom	x	y	z	$U_{\rm iso}$ (Å^2)
Fe1	0	0	0	0.0114(2)
Fe2	0.5	0	0	0.0114
K	0.7499(3)	0.56149(11)	−0.01970(19)	0.0314(5)
C1	−0.0322(6)	0.8580(9)	0.7744(9)	0.0051(6)
C2	−0.0318(6)	0.2110(9)	0.8542(9)	0.0051
C3	0.8123(6)	−0.0004(10)	0.0619(9)	0.0051
N1	−0.0437(5)	0.7540(7)	0.6584(7)	0.0051
N2	0.9485(5)	0.3353(7)	0.7609(7)	0.0051
N3	0.7045(5)	−0.0312(7)	0.0913(7)	0.0051

Table S7: Crystallographic parameters for $P2_1/n$ structure of $K_{1.9736(18)}Mn[Fe(CN)_6]$ at room temperature from the fit the data in Fig. 4 of the main text.

a (Å)	10.0991(15)			
b (Å)	7.3349(4)			
c (Å)	6.948(4)			
β (°)	90.0118(5)			
V (Å^3)	514.75(9)			
Z	4			
Atom	x	y	z	$U_{\rm iso}$ (Å^2)
Fe	0	0	0	0.01322(11)
Mn	0.5	0	0	0.01322
K1	0.7517(2)	0.56517(6)	−0.02040(10)	0.0343(3)
C1	−0.0344(5)	0.8550(6)	0.7713(6)	0.0174(4)
C2	−0.0356(5)	0.2136(6)	0.8554(6)	0.0174
C3	0.8174(4)	−0.0133(7)	0.0667(6)	0.0174
N1	−0.0517(4)	0.7642(5)	0.6491(5)	0.0174
N2	0.9429(4)	0.3412(5)	0.7640(5)	0.0174
N3	0.7070(3)	−0.0294(5)	0.1032(5)	0.0174

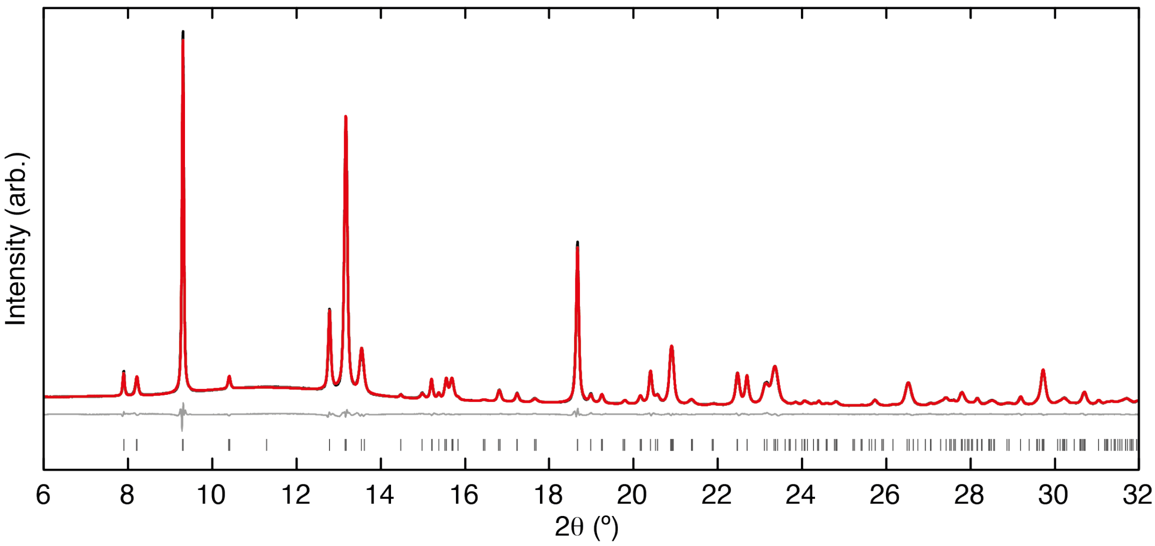

Figure S6: Rietveld fit to the X-ray powder diffraction pattern measured for $K_2Cd[Fe(CN)_6]$ at room temperature. Raw data in black, fit in red, and corresponding difference curve in grey (data – fit) is offset below the data. Tick marks denote reflection positions.

Table S8: Crystallographic parameters for $P2_1/n$ structure of $K_{1.954(2)}Cd[Fe(CN)_6]$ at room temperature from the fit the data in Fig. S6.

a (Å)	10.176(12)			
b (Å)	7.413(3)			
c (Å)	6.998(3)			
β (°)	90.1474(13)			
V (Å^3)	527.9(7)			
Z	4			
Atom	x	y	z	U_{iso} (Å^2)
Fe	0	0	0	0.01334(11)
Cd	0.5	0	0	0.01334
K	0.75059(12)	0.57004(8)	−0.02145(19)	0.0394(4)
C1	−0.0366(5)	0.8515(6)	0.7656(6)	0.0194(5)
C2	−0.0445(5)	0.2203(6)	0.8543(6)	0.0194
C3	0.8114(4)	−0.0077(9)	0.0725(6)	0.0194
N1	−0.0533(4)	0.7770(5)	0.6561(5)	0.0194
N2	0.9378(4)	0.3377(5)	0.7781(5)	0.0194
N3	0.7166(3)	−0.0404(5)	0.1181(5)	0.0194

Variable-temperature $K_2Mn[Fe(CN)_6]$ data: refinement details

Our refinement strategy for $K_2Mn[Fe(CN)_6]$ differed from the general approach for other samples due to its superior crystallinity and thermal stability, which allowed a more detailed refinement.

For $K_2Mn[Fe(CN)_6]$ a sequential Rietveld refinement with the single phase $P2_1/n$ model was used for the temperature range 300–795 K after which point monoclinic peaks disappear and the relative intensities of the 200 & 110 (and 400 & 220) peaks begin to change with the emergence of a tetragonal phase, to which we assign the label **T**′. A reliable model for **T**′ was acquired by fitting a pattern measured at 803 K using a two-phase Rietveld refinement. At this temperature, the **T**′ phase was present at a sufficiently high concentration to allow reliable fitting of the peak intensities. Below 795 K the phase fraction for **T**′ was lower than 5% and so this phase was not included in any such refinements. For the region 795–803 K the **T**′ phase was at low concentration and with many overlapping peaks, and so atomic coordinates were fixed.

For the region 803–853 K, the **T**′ phase was allowed to refine freely and the monoclinic phase had its atomic positions fixed for the lighter C and N atoms.

The region 853–884 K contains all three of the phases observed over the full temperature range. The challenge of refining all three phases meant that more constraints had to be introduced. The monoclinic peaks ceased to move in 2θ so only the scale was allowed to refine. The anisotropic displacement parameters for each of the tetragonal phases were fitted parametrically using linear fits to the values that emerged from refinements carried out over the 822–853 K (**T**′) and 920–1000 K (**T**) regions, where the corresponding phases are the dominant phase present. Furthermore, the atomic positions for the **T** phase were fixed to values obtained in a refinement at 898 K.

886 K is the lowest temperature at which the monoclinic phase has entirely disappeared, and therefore no slide distortion is present in $K_2Mn[Fe(CN)_6]$ for temperatures $T > 886$ K. By 898 K **T** is the dominant phase and therefore allowed to refine freely, with restrictions now imposed on the atomic coordinates of **T**′. At higher temperatures, the **T**′ phase is gradually consumed, leaving the **T** phase as the only PBA phase present by 980 K. This phase is stable up to 1000 K with only a small amount of decomposition evident from the formation of crystalline MnO.

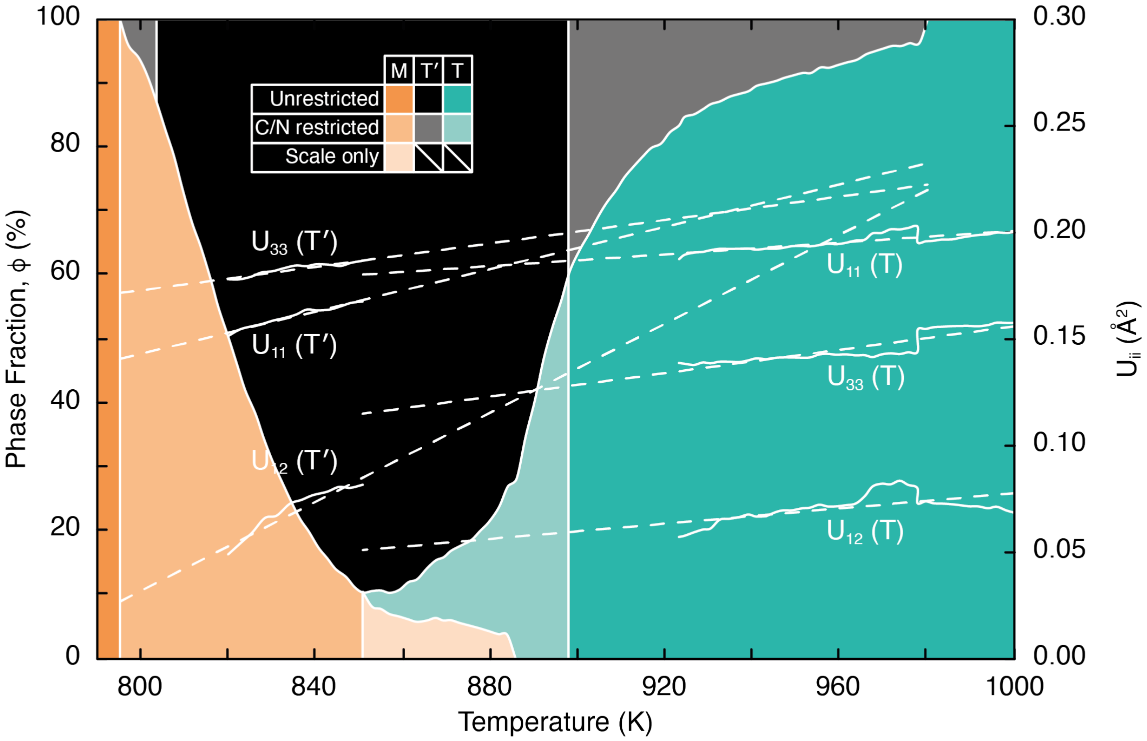

Figure S7: Map of phase fractions for $K_2Mn[Fe(CN)_6]$ between 790 and 1000 K. Monoclinic phase in orange, **T**′ in black and **T** in teal. The anisotropic thermal displacement parameters for each tetragonal phase are plotted on the secondary axis with fits to refined regions illustrated.

Table S9: Crystallographic parameters for $P2_1/n$ structure of $K_2Mn[Fe(CN)_6]$ at 795 K.

a (Å)	10.320(3)			
b (Å)	7.320(8))			
c (Å)	7.206(8)			
β (°)	89.924(3)			
V (Å^3)	545(2)			
Z	4			
Atom	x	y	z	U_{iso} (Å^2)
Fe	0	0	0	0.0333(3)
Mn	0.5	0	0	0.0333
K	0.7528(6)	0.5262(4)	−0.0079(5)	0.1346(6)
C1	−0.0394(12)	0.8421(15)	0.7876(15)	0.0512(9)
C2	−0.0202(12)	0.1968(15)	0.8469(15)	0.0512
C3	0.8175(9)	0.0047(19)	0.0173(17)	0.0512
N1	−0.0318(9)	0.7711(11)	0.6605(11)	0.0512
N2	0.9822(9)	0.3424(11)	0.7586(11)	0.0512
N3	0.7106(9)	−0.0222(12)	0.0813(11)	0.0512

Table S10: Crystallographic parameters for **T**′ $P4/mnc$ structure of $K_2Mn[Fe(CN)_6]$ at 803 K.

a (Å)	7.308(5)						
c (Å)	10.276(5)						
V (Å^3)	548.9(9)						
Z	4						
Atom	x	y	z	U_{11} (Å^2)	U_{33} (Å^2)	U_{12} (Å^2)	U_{iso} or U_{eq} (Å^2)
Fe	0	0	0				0.0380(15)
Mn	0	0	0.5				0.0380
K	0	0.5	0.25	0.1590	0.1822	0.0650	0.1667
C1	0.802(3)	0.169(3)	0				0.087(5)
C2	0	0	0.149(3)				0.087
N1	0.656(2)	0.246(2)	0				0.087
N2	0	0	0.281(3)				0.087

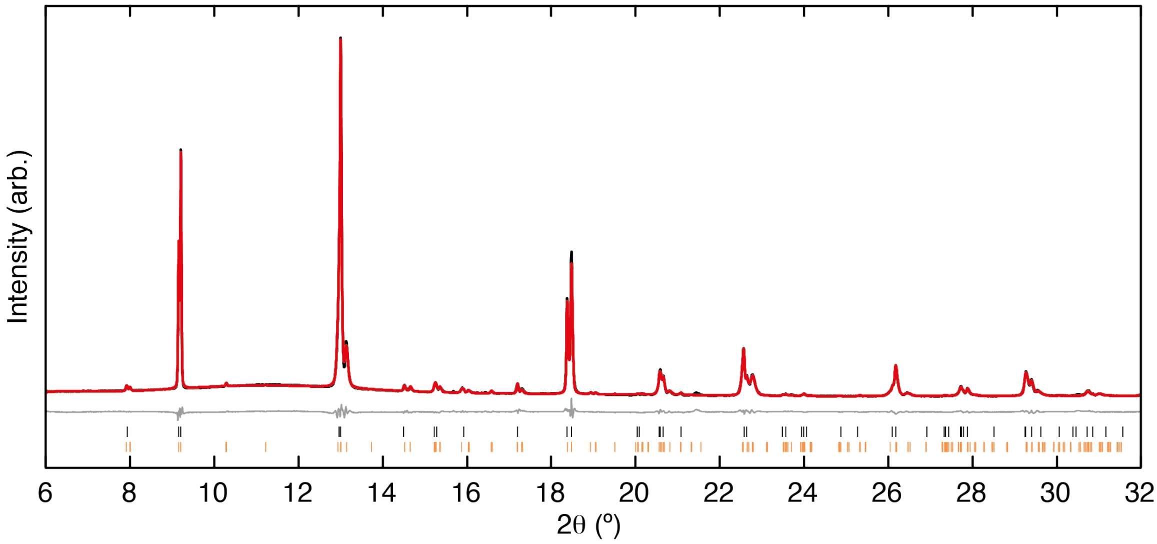

Figure S8: Rietveld fit to the X-ray powder diffraction pattern measured for $K_2Mn[Fe(CN)_6]$ at 803K. Raw data in black, fit in red, and corresponding difference curve in grey (data – fit) is offset below the data. Tick marks denote reflection positions for the monoclinic (orange) and **T**′ (black) phases.

Table S11: Crystallographic parameters for **T**′ $P4/mnc$ structure of $K_2Mn[Fe(CN)_6]$ at 898 K.

a (Å)	7.321(6)						
c (Å)	10.311(6)						
V (Å^3)	552.7(10)						
Z	4						
Atom	x	y	z	U_{11} (Å^2)	U_{33} (Å^2)	U_{12} (Å^2)	U_{iso} or U_{eq} (Å^2)
Fe	0	0	0				0.0571(13)
Mn	0	0	0.5				0.0571
K	0	0.5	0.25	0.1801	0.1938	0.1080	0.1847
C1	0.795(2)	0.156(2)	0				0.0747(3)
C2	0	0	0.1764(15)				0.0747
N1	0.6693(15)	0.2408(15)	0				0.0747
N2	0	0	0.2904(15)				0.0747

Table S12: Crystallographic parameters for **T** $P4/mnc$ structure of $K_2Mn[Fe(CN)_6]$ at 898 K.

a (Å)	7.299(6)						
c (Å)	10.387(6)						
V (Å^3)	553.4(10)						
Z	4						
Atom	x	y	z	U_{11} (Å^2)	U_{33} (Å^2)	U_{12} (Å^2)	U_{iso} or U_{eq} (Å^2)
Fe	0	0	0				0.0280(5)
Mn	0	0	0.5				0.0280
K	0	0.5	0.25	0.1837	0.1226	0.0556	0.1633
C1	0.7917(19)	0.1564(19)	0				0.120(3)
C2	0	0	0.1925(17)				0.120
N1	0.6628(14)	0.2233(14)	0				0.120
N2	0	0	0.2939(16)				0.120

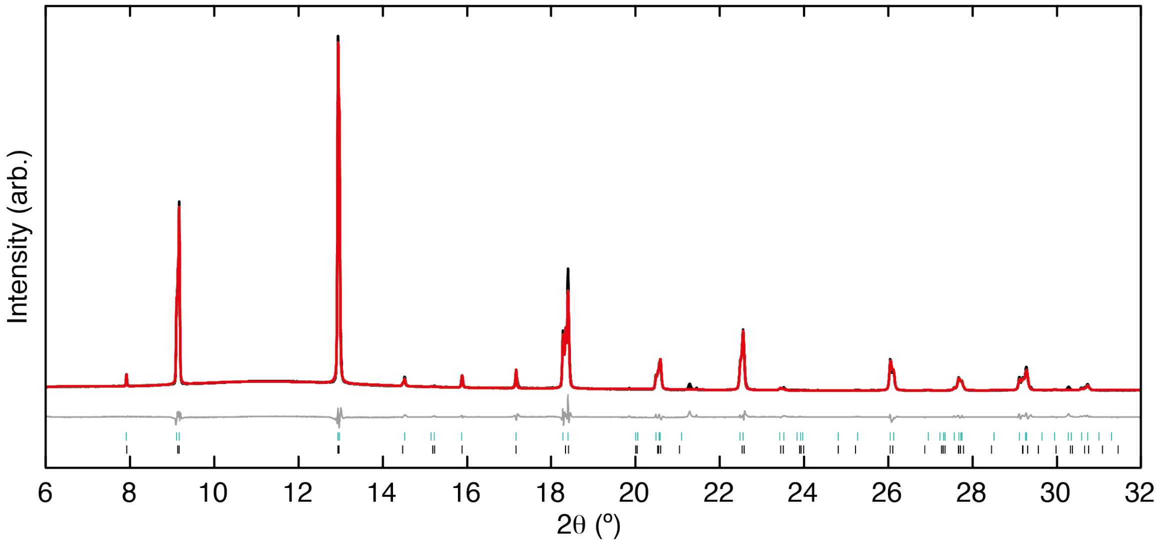

Figure S9: Rietveld fit to the X-ray powder diffraction pattern measured for $K_2Mn[Fe(CN)_6]$ at 898K. Raw data in black, fit in red, and corresponding difference curve in grey (data – fit) is offset below the data. Tick marks denote reflection positions for the **T**′ (black) and **T** (teal) phases.

Table S13: Crystallographic parameters for **T** $P4/mnc$ structure of $K_2Mn[Fe(CN)_6]$ at 1000 K.

a (Å)	7.325(2)						
c (Å)	10.404(3)						
V (Å^3)	558.2(4)						
Z	4						
Atom	x	y	z	U_{11} (Å^2)	U_{33} (Å^2)	U_{12} (Å^2)	U_{iso} or U_{eq} (Å^2)
Fe	0	0	0				0.0408(3)
Mn	0	0	0.5				0.0408
K	0	0.5	0.25	0.2014	0.1572	0.0776	0.1867
C1	0.8041(18)	0.1604(18)	0				0.1079(15)
C2	0	0	0.1888(14)				0.1079
N1	0.6746(12)	0.2418(12)	0				0.1079
N2	0	0	0.2911(13)				0.1079

Variable-temperature $K_2M[Fe(CN)_6]$ (M = Cd, Fe, Co, Ni) data: refinement details

A similar but simplified approach was taken for the other $K_2M[Fe(CN)_6]$ samples. A sequential Rietveld refinement with the single phase $P2_1/n$ model used. At the point where the relative intensities of the 200 & 110 (and 400 & 220) peaks began to change, a tetragonal phase was introduced.

An .xlsx file containing all exported parameters from the VT refinements is included.

Table S14: Thermal Expansion of $K_2M[Fe(CN)_6]$ calculated using $PASCal$.[S7] α_1 is the thermal expansion along the b-axis. α_2 and α_3 correspond to approximately the a- and c-axes, respectively. T_c and T_d are transition temperatures and decomposition temperatures, respectively. T_d was assigned at the first pattern where crystalline peaks for a new non-PBA phase were first observed before growing progressively with heating, while the total phase fraction for the PBA decreased.

M	α_1 (MK^{-1})	α_2 (MK^{-1})	α_3 (MK^{-1})	α_V (MK^{-1})	T_c (K)	T_d (K)
Ni	−6.32(14)	47.7(5)	87.6(10)	130.9(14)	518.1	619.7
Co	−1.6(2)	44.2(3)	79.3(8)	123.3(9)	572.8	731.0
Fe	−1.7(2)	45.9(3)	74.9(7)	120.5(7)	599.3	778.2
Mn	−4.1(3)	44.3(3)	75.3(8)	117.6(9)	797.0	974.2
Cd	−0.7(4)	38.9(4)	64.4(5)	104.9(11)	841.0	881.8

5 References

(S1) Wang, K.; Jacobsen, S. B. An estimate of the Bulk Silicate Earth potassium isotopic composition based on MC-ICPMS measurements of basalts. *Geochim. Cosmochim. Acta* **2016**, *178*, 223–232.

(S2) Coelho, A. A; *TOPAS-Academic, version 6 (computer software)*, Coelho Software, Brisbane.

(S3) Stokes, H. T.; Hatch, D. M.; Campbell, B. J. *ISODISTORT*, ISOTROPY Software Suite, iso.byu.edu.

(S4) Campbell, B. J.; Stokes, H. T.; Tanner, D. E.; Hatch, D. M. ISODISPLACE: A web-based tool for exploring structural distortions. *J. Appl. Cryst.* **2006**, 39, 607–614.

(S5) Cattermull, J.; Pasta, M.; Goodwin, A. L. Structural Complexity in Prussian Blue Analogues. *Mater. Horiz.* **2021**, *8* 3178–3186.

(S6) Stephens, P.W.; *J. Appl. Cryst.* **1999**, *32*, 281–289.

(S7) Cliffe, M. J.; Goodwin, A. L. $PASCal$: A Principal Axis Strain Calculator for Thermal Expansion and Compressibility Determination. *J. Appl. Cryst.* **2012**, *45*, 1321–1329.
